# Endosomal recycling tubule scission and integrin recycling involve the membrane curvature-supporting protein LITAF

**DOI:** 10.1242/jcs.258549

**Published:** 2021-08-03

**Authors:** Lydia Wunderley, Ling Zhang, Rebecca Yarwood, Wenxia Qin, Martin Lowe, Philip Woodman

**Affiliations:** Faculty of Biology Medicine and Health, Manchester Academic and Health Science Centre, University of Manchester, Manchester M13 9PT, UK

**Keywords:** LITAF, Recycling endosome, Rab11, EHD, SNX, Charcot Marie Tooth

## Abstract

Recycling to the cell surface requires the scission of tubular membrane intermediates emanating from endosomes. Here, we identify the monotopic membrane protein LPS-induced TNF-activating factor (LITAF) and the related protein cell death involved p53 target 1 (CDIP1) as novel membrane curvature proteins that contribute to recycling tubule scission. Recombinant LITAF supports high membrane curvature, shown by its ability to reduce proteoliposome size. The membrane domains of LITAF and CDIP1 partition strongly into ∼50 nm diameter tubules labelled with the recycling markers Pacsin2, ARF6 and SNX1, and the recycling cargoes MHC class I and CD59. Partitioning of LITAF into tubules is impaired by mutations linked to Charcot Marie Tooth disease type 1C. Meanwhile, co-depletion of LITAF and CDIP1 results in the expansion of tubular recycling compartments and stabilised Rab11 tubules, pointing to a function for LITAF and CDIP1 in membrane scission. Consistent with this, co-depletion of LITAF and CDIP1 impairs integrin recycling and cell migration.

## Abstract (precis)

**Summary:** LITAF is an endosomal monotopic membrane protein that is mutated in Charcot Marie Tooth disease type 1C. We show that LITAF is important for endosomal recycling through supporting the scission of recycling tubules.

## INTRODUCTION

Cells modulate their complement of surface membrane proteins by the process of endosomal sorting. When internalised surface proteins reach the early endosome, some are transported via the multivesicular body (MVB) to the lysosome and degraded, while others recycle to be re-exposed on the surface (Cullen and Steinberg, 2018; Naslavsky and Caplan, 2018).

Recycling pathways are pleiomorphic (Cullen and Steinberg, 2018; Goldenring, 2015; Mayor et al., 2014; Naslavsky and Caplan, 2018), reflecting the diversity of recycled cargo. Many cargoes recycle via tubular intermediates that arise from sorting endosomes and coalesce in the perinuclear region. Several cargoes partition into the endosomal recycling compartment (ERC), which is enriched for the small GTPase Rab11 (herein referring collectively to Rab11a and Rab11b) (Goldenring, 2015). The ERC is functionally interconnected with another recycling compartment, the tubular recycling endosome (TRE) (Cullen and Steinberg, 2018; Dhawan et al., 2020; Naslavsky et al., 2006). The TRE is controlled by ARF6 (Radhakrishna and Donaldson, 1997) and Rab10 (Etoh and Fukuda, 2019; Farmer et al., 2021).

Generation of recycling tubules requires membrane curvature proteins. For example, SNX-BAR family members help form tubules to recycle a range of cargoes (Cullen and Steinberg, 2018; van Weering et al., 2012; Weeratunga et al., 2020). Meanwhile, the F-BAR protein Pacsin2 (also known as syndapin 2) (Shimada et al., 2010; Wang et al., 2009) cooperates with MICAL-L1 (Giridharan et al., 2013; Sharma et al., 2009) to control TRE morphogenesis (Caplan et al., 2002; Naslavsky and Caplan, 2011).

An imperative of maintaining compartmental boundaries during endosomal recycling is that tubules undergo scission at the endosome before they expand far enough to contact and fuse with their target membrane. The family of EHD ATPases, notably EHD1, couple nucleotide hydrolysis to membrane scission (Deo et al., 2018; Kamerkar et al., 2019; Naslavsky and Caplan, 2011). *In vitro* studies show that ATP hydrolysis promotes EHD1 oligomerization at membranes, supporting membrane deformation and leading to membrane scission (Daumke et al., 2007; Deo et al., 2018). EHD1 is recruited to TRE tubules by interactions with MICAL-L1 and Pacsin2 (Braun et al., 2005; Sharma et al., 2009), and recent studies show that EHD1 is also recruited to SNX-BAR tubules (Dhawan et al., 2020; Solinger et al., 2020). However, the mechanisms underlying the morphogenesis of endosomal recycling tubules and membrane scission remain only partially understood.

LPS-induced TNF-activating factor [LITAF; also known as small integral membrane protein of the late endosome (SIMPLE)] (Myokai et al., 1999) localises to endosomes (Eaton et al., 2011; Moriwaki et al., 2001), and defines a novel class of monotopic integral membrane protein that is anchored within the membrane via a conserved C-terminal region containing a zinc finger (Ho et al., 2016; Qin et al., 2016). Mutations in LITAF give rise to the peripheral neuropathy, Charcot Marie Tooth disease type 1C (CMT1C) (Azzedine et al., 2012; Street et al., 2003). However, although LITAF disease mutations induce endosomal vacuolation (Edgar et al., 2020; Lee et al., 2012; Zhu et al., 2013), the mechanisms underlying CMT1C remain elusive. Here, we identify LITAF as a new membrane curvature protein that regulates the morphogenesis of recycling endosomes and integrin trafficking by supporting the scission of recycling tubules.

## RESULTS

### LITAF contains a conserved membrane curvature domain that partitions into tubular recycling endosomes

**Fig. 1.**
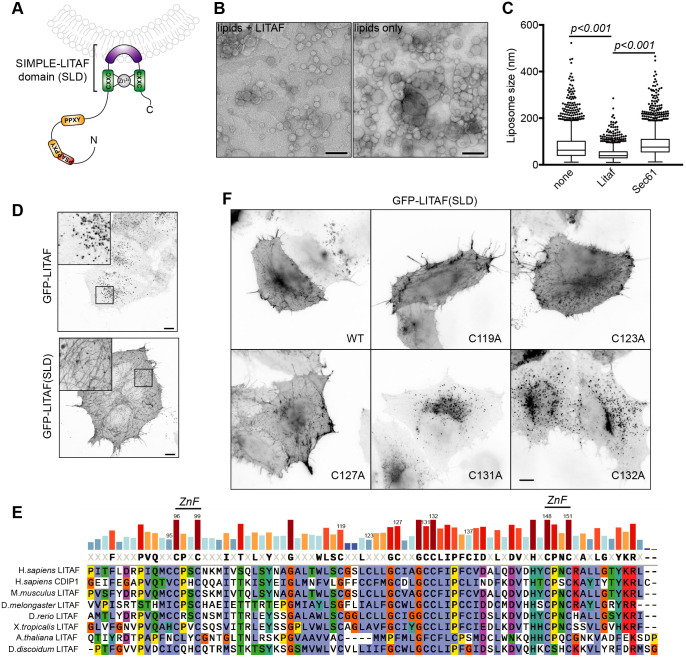
**LITAF supports membrane curvature.** (A) Diagram showing the membrane topology of LITAF. PPXY, Nedd4-binding motif; PTAP, ESCRT-I-binding motif. (B) Representative EM images of liposomes reconstituted with (left panel) or without (right panel) recombinant LITAF. (C) Size analysis of liposomes made without protein (*n*=2732; six experiments), or with either His_6_-LITAF (*n*=1900 measurements from three independent experiments) or His_6_–Sec61β (*n*=2014; three experiments). Results represented as Tukey box plot with outliers shown. *P*-values calculated with a Kolmogorov–Smirnov test. (D) Representative confocal microscopy images of HeLaM cells transfected with GFP–LITAF or GFP–LITAF(SLD (E) ClustalX alignment of SIMPLE-LITAF domains. (F) Representative widefield microscopy images of HeLaM cells transfected with the indicated GFP–LITAF(SLD) mutants. Scale bars: 10 µm.

LITAF defines a class of monotopic integral membrane protein that associates with endosomal membranes via a conserved C-terminal SIMPLE-LITAF domain (SLD). The SLD comprises a zinc finger that harbours a 22-amino-acid hydrophobic membrane anchor (Ho et al., 2016; Qin et al., 2016) (Fig. 1A). Since other monotopic integral membrane proteins, such as caveolin and reticulons, support membrane curvature (Goyal and Blackstone, 2013; Parton and del Pozo, 2013), we explored whether LITAF might act as a membrane curvature protein within the endocytic pathway.

The ability of reticulons to induce membrane curvature is manifested in them reducing the size of liposomes generated from lipid–protein–detergent mixtures, thus favouring the production of highly curved membranes (Hu et al., 2008; Khaminets et al., 2015). Negative-stain electron microscopy (EM) showed that liposomes made in the presence of His_6_–LITAF were significantly smaller (median diameter, 39.3 nm; 75% percentile, 55.9 nm) than those made without protein (median diameter, 63.0 nm; 75% percentile, 100.6 nm) or with His_6_–Sec61β as a control C-terminally anchored protein (median diameter, 75.6 nm; 75% percentile, 109.1 nm) (Fig. 1B,C). Hence, LITAF resembles reticulons in its impact on membrane curvature in a reconstituted system.

Membrane curvature domains often partition to tubular compartments (Voeltz et al., 2006). In HeLaM cells, GFP–LITAF(SLD) or StrepTag–LITAF(SLD) decorated tubules strongly while also labelling the plasma membrane clearly (Fig. 1D; Fig. S1A,B). In contrast, full-length (FL) LITAF localised strongly to vesicular compartments (Fig. 1D; Fig. S1A,B), previously identified as early and late endosomes (Ho et al., 2016; Lee et al., 2012; Moriwaki et al., 2001; Qin et al., 2016; Zhu et al., 2013). However, close examination showed that some exogenous (Fig. S1A,B) and endogenous (Fig. S1D) LITAF also labelled tubules weakly. Hence, partitioning into tubules is an inherent behaviour of the SLD, and is modulated within FL LITAF. Partitioning to tubules is a conserved feature of the SLD, since the SLD of cell death involved p53 target 1 (CDIP1), the other LITAF family protein expressed in humans (Brown et al., 2007; Qin et al., 2016), also localised strongly to tubules (Fig. S1A,C), whereas FL CDIP1 localised to vesicular compartments (Fig. S1A,C).

Mutating conserved ligand-binding cysteine residues in the Zn^2+^-finger prevented the membrane localization of GFP–LITAF(SLD) (Fig. S1E), as was seen for FL LITAF (Ho et al., 2016; Qin et al., 2016). The hydrophobic insertion within the SLD contains conserved cysteine residues (C130 and C131) that are also found in CDIP1 (Fig. 1E). C130A and C131A mutants localised strongly to endosomes (Fig. 1F; Fig. S1F). In contrast, when other cysteine residues (C119A, C123A or C127A) were mutated, the SLD behaved like wild-type (WT) GFP–LITAF(SLD) (Fig. 1F). Hence, conserved cysteine residues in the membrane anchor allow LITAF to partition into high-curvature membrane tubules.

**Fig. 2.**
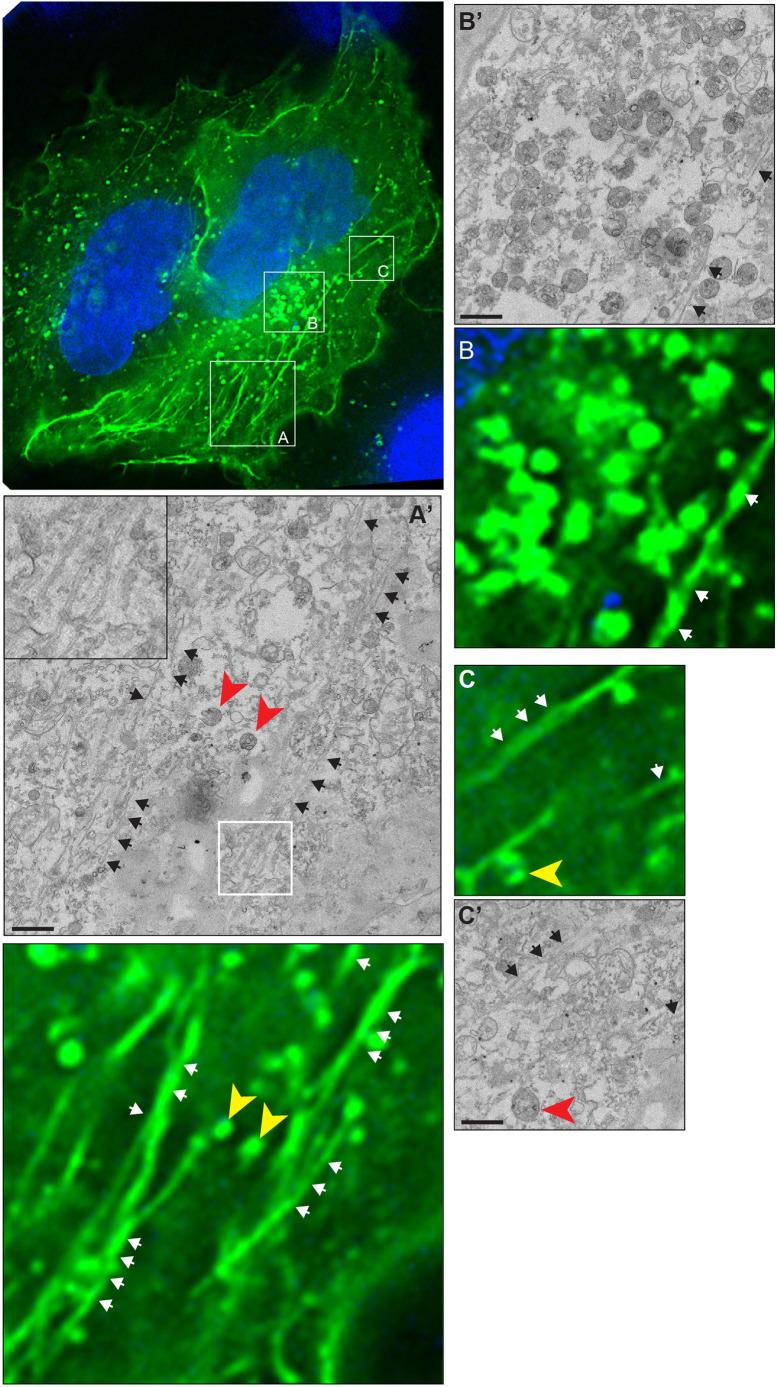
**Correlative light electron microscopy identifies LITAF SLD tubules.** Correlative light-EM microscopy of cells expressing GFP–LITAF(SLD). Only fluorescence image is shown in the main panel; boxed areas from the main panel (A–C) are shown magnified ×5 (A′–C′), with a further inset for panel A′. Arrows and arrowheads from EM images highlight tubules and MVBs, respectively, as markers, and are transposed onto fluorescence images (with minor shifts to account for sample shrinkage). Scale bars: 1 µm. Tubule diameters were assessed from 191 random cross-sectional measurements.

**Fig. 3.**
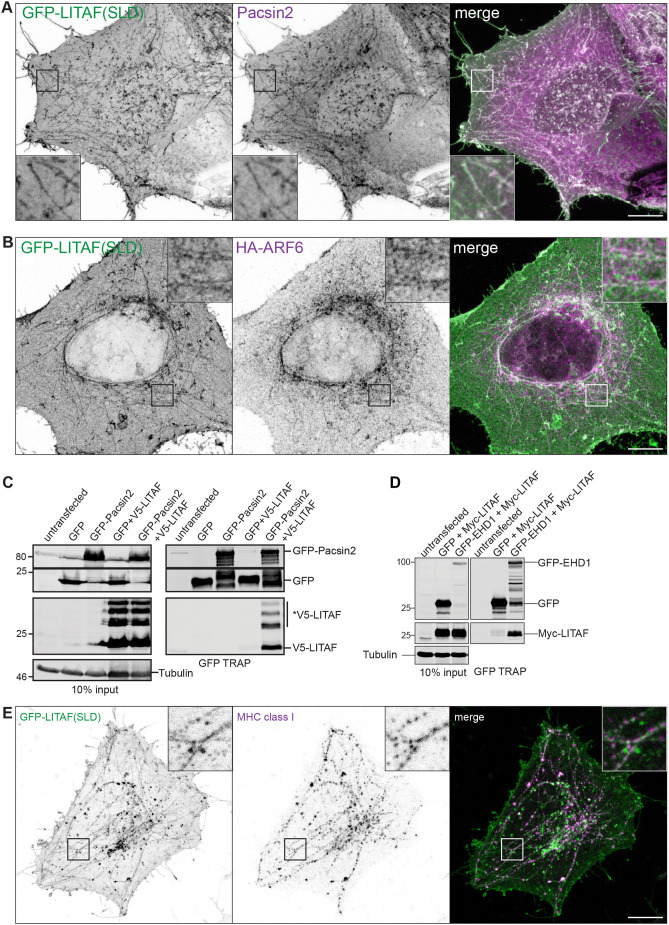
**LITAF localises to recycling endosomal tubules.** (A) Representative confocal microscopy images of HeLaM cells transfected with GFP–LITAF(SLD) and stained for Pacsin2. (B) Representative confocal microscopy images of HeLaM cells co-transfected with GFP-LITAF(SLD) and HA-tagged ARF6. (C) HEK293 cells were co-transfected with GFP or GFP–Pacsin2, together with V5–LITAF as indicated. Cell lysates were incubated with GFP-Trap. An asterisk denotes higher molecular mass forms of V5–LITAF. (D) HEK293 cells were co-transfected with GFP or GFP–EHD1, together with Myc-LITAF as indicated. Cell lysates were incubated with GFP-Trap. Blots are representative of three experiments. (E) Representative confocal microscopy images of HeLaM cells transfected with GFP–LITAF(SLD) and pulse-labelled for 30 min with anti-MHC class I antibody. Scale bars: 10 µm.

Correlative light electron microscopy (CLEM) confirmed that GFP–LITAF(SLD) associated with linear membrane tubules of uniform diameter (54.2 nm, median; 95% confidence limits 52.1–56.0 nm) as well as with endosomes (Fig. 2). These tubules resemble the exaggerated tubular recycling endosome (TRE) induced in HeLa cells upon expression of an ARF6 dominant-negative mutant, overexpression of Pacsin2, or either overexpression or depletion of the scission ATPase EHD1 (Braun et al., 2005; Caplan et al., 2002; Radhakrishna and Donaldson, 1997; Sharma et al., 2009). Although some GFP–LITAF(SLD) tubules did not label with antibodies for TRE markers, nearly all Pacsin2-positive tubules (Fig. 3A; 90%; data from 31 cells in three independent experiments) and MICAL-L1-positive tubules (96%; 35 cells; three experiments) contained GFP–LITAF(SLD). LITAF(SLD) also labelled tubules containing HA-tagged ARF6 (Fig. 3B). Furthermore, co-expressing HA–ARF6^T44N^, which prevents nucleotide exchange (Macia et al., 2004), enhanced the tubular morphology of GFP–LITAF(SLD)-labelled areas, and also to some extent those of FL GFP–LITAF (Fig. S2A). GFP–LITAF(SLD) partitioned into tubules in Vero, U2OS and COS7 cells (Fig. S2B). Indeed, Pacsin2 tubules were rarely seen in untransfected Vero cells but this protein localised to short GFP–LITAF(SLD) tubules in transfected cells (Fig. S2C), suggesting that the LITAF SLD might be able to induce a tubular recycling compartment. Further establishing a link between LITAF and the TRE, epitope-tagged LITAF co-immunoprecipitated with GFP–Pacsin2 (Fig. 3C) and with GFP–EHD1 (Fig. 3D).

Several clathrin-independent cargoes including MHC class I, CD59 and CD98 (a heterodimer of SLC3A2 and SLC7A5) recycle through the TRE (Caplan et al., 2002; Naslavsky et al., 2004; Radhakrishna and Donaldson, 1997). Many GFP–LITAF(SLD) tubules were labelled with internalised anti-MHC class I (Fig. 3E) and anti-CD59 antibodies (Fig. S3A). Hence, LITAF(SLD) labels a functional TRE. SNX1 generates a recycling intermediate that overlaps functionally with the TRE (Cullen and Steinberg, 2018; Dhawan et al., 2020; Solinger et al., 2020). SNX1 tubules were less pronounced than TRE tubules in HeLa cells, but also often contained GFP–LITAF(SLD) (Fig. S3B). Finally, a subset of GFP–LITAF(SLD) tubules labelled with an antibody (Hammond et al., 2009) and GFP sensor (Hammond et al., 2014) against phosphatidylinositol-4-phosphate (PtdIns4P), a lipid produced transiently during endosomal recycling (Henmi et al., 2016; Jović et al., 2009; Ketel et al., 2016; Wallroth and Haucke, 2018) (Fig. S3C,D). The partitioning of LITAF(SLD) to tubules is selective for recycling endosome compartments, since GFP–LITAF(SLD) did not colocalise with TGN46 (TGOLN2)-labelled tubules, induced by treating cells with Brefeldin A (Fig. S3E). Overall, these data underscore a link between LITAF and endosomal recycling.

### LITAF CMT1C disease mutants reduce partitioning to the TRE

**Fig. 4.**
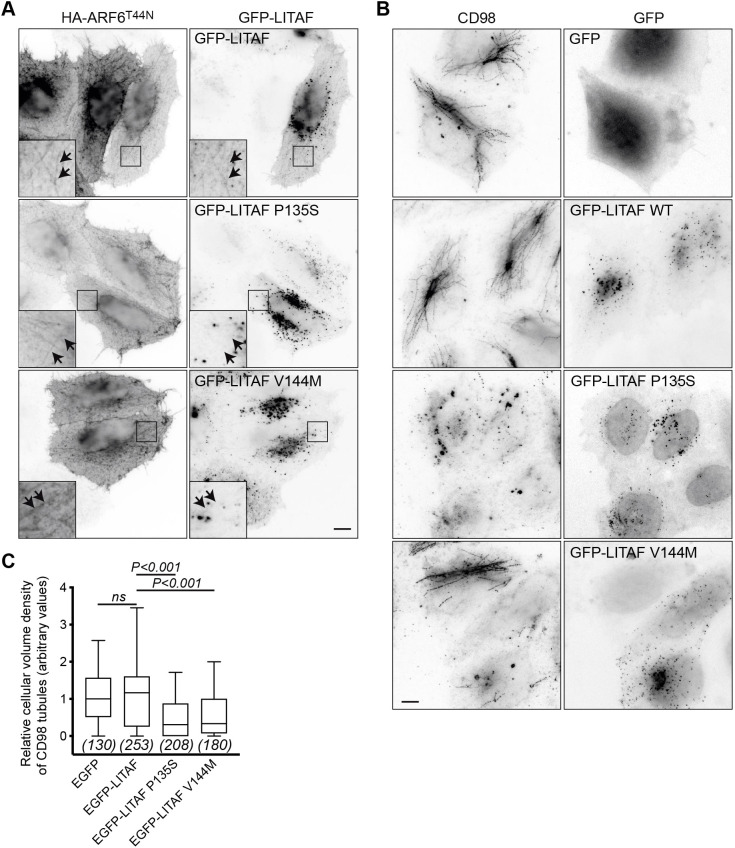
**LITAF CMT1C mutants reduce partitioning to the TRE.** (A) Representative widefield images HeLaM cells co-transfected with HA–ARF6^T44N^ and WT or the indicated mutants of GFP–LITAF. (B) Representative widefield images HeLaM cells transfected with the indicated constructs were pulse labelled with anti-CD98 antibody for 3 h at 37°C. (C) Quantification of the effect of LITAF mutants on CD98 tubule density. Results represented as Tukey box plot. Data were combined from three independent experiments, total cell numbers shown in brackets. Statistical analysis by one-way ANOVA [*F*=45.29; d.f.=764]; ns, not significant. Scale bars: 10 µm.

We next examined whether CMT1C mutations affect the distribution of LITAF. As FL LITAF partitions modestly to recycling tubules, we co-expressed ARF6^T44N^ to stabilise recycling tubules and thus enhance the partitioning of LITAF into these. Under these conditions, some GFP–LITAF distributed to TRE tubules. However, two CMT1C mutants, GFP–LITAF(P135S) and GFP–LITAF(V144M), remained strongly associated with endosomes (Fig. 4A). Supporting these data, neither GFP–LITAF(SLD)(P135S) nor GFP–LITAF(SLD)(V144M) localised to ARF6^T44N^ tubules, but instead labelled endosomes (Fig. S4).

CMT(1C) is autosomal dominant (Street et al., 2003). To explore whether CMT(1C) mutations exhibit a gain-of-function effect on the TRE, we examined how they affected the distribution of internalised CD98, a cargo that partitions strongly to the TRE (Eyster et al., 2009). In cells expressing either GFP–LITAF(P135S) or GFP–LITAF(V144M), internalised CD98 redistributed from tubules to vacuolar structures (Fig. 4B,C). Hence, these CMT1C mutations impair the ability of LITAF to associate with high-curvature recycling tubules and reduce cargo partitioning into these tubules.

### LITAF and CDIP1 control the dynamics of membrane tubule scission and maintain compartmental boundaries within the endocytic pathway

**Fig. 5.**
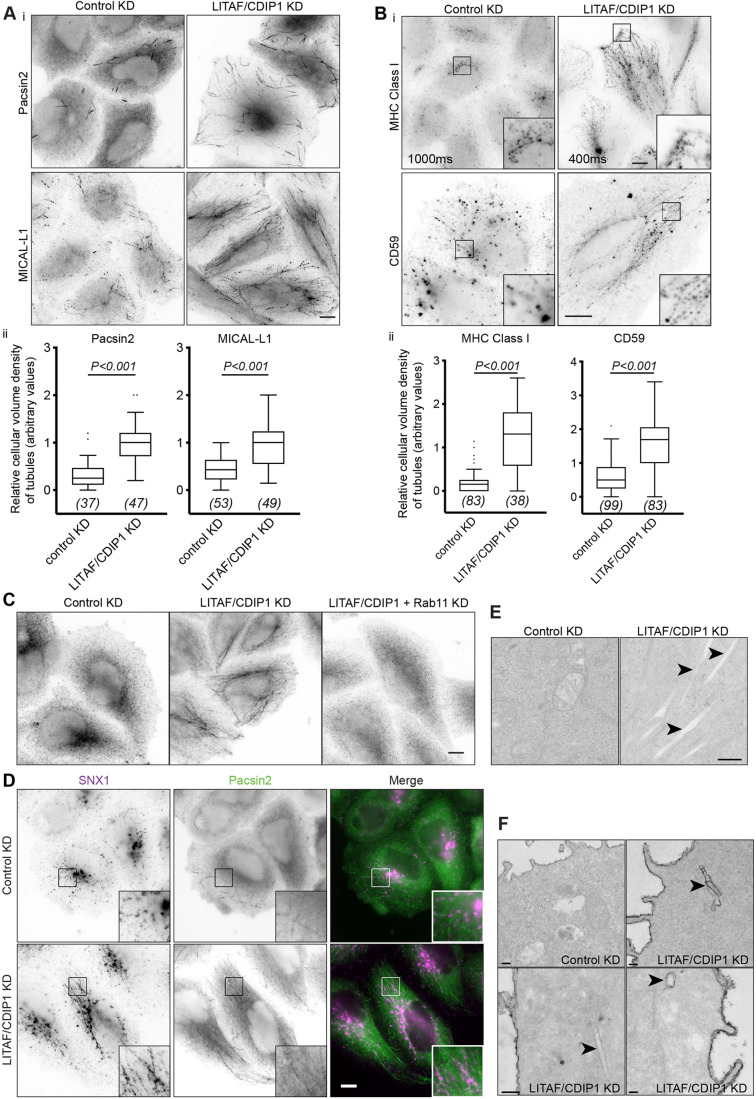
**LITAF/CDIP1 control the tubular recycling endosome.** (Ai) Representative widefield images control or LITAF/CDIP1-depleted (KD) cells stained for Pacsin2 or for MICAL-L1. (Aii) Quantification of Pacsin2 and MICAL-L1 tubule density. Data combined from three independent experiments, total cell numbers shown in brackets. (Bi) Representative widefield images of control or LITAF/CDIP1-depleted HeLaM cells pulse-labelled with anti-MHC class I antibody or anti-CD59 antibody for 30 min at 37°C. Scale bar: 10 µm. (Bii) Quantitation of anti-MHC class I and anti-CD59-labelled tubule density. Quantitative results represented as Tukey box plot. *P*-values calculated with a Kolmogorov–Smirnov test. (C) Representative widefield images of control, LITAF/CDIP1-knockdown, or LITAF/CDIP1- and Rab11 co-depleted HeLaM cells, fixed and stained for Rab11. (D) Representative widefield images of control or LITAF/CDIP1-depleted cells stained for Pacsin2 and SNX1. (E) Representative EM images of control or LITAF/CDIP1-depleted cells. (F) Representative EM images of control or LITAF/CDIP1-depleted cells fixed in the presence of Ruthenium Red and processed for EM using conditions that minimally stained membranes that did not encounter ruthenium Red. Scale bars: 10 µm (A–D); 0.2 µm (E,F). Arrowheads highlight tubules.

Our data establish that LITAF associates with tubular recycling compartments, notably the TRE. The TRE expands when the ATPase EHD1 is depleted (Cai et al., 2013), reflecting reduced tubule scission. We examined how the TRE and potentially other recycling compartments were affected when LITAF activity is removed. We co-depleted CDIP1 because of its likely functional overlap with LITAF (herein referred to as LITAF/CDIP1-depleted cells), given their similar membrane topology and localisation (Qin et al., 2016), and association with each other (Fig. S5A). In LITAF/CDIP1-depleted cells (Fig. S5B), the density of tubules positive for Pacsin2 and MICAL-L1 increased markedly (Fig. 5A), which is very similar to the effects of EHD1 depletion (Cai et al., 2013). Exaggerated TRE tubules contained recycling cargo, since the density of tubules labelled with internalised MHC class I and CD59 also increased markedly upon LITAF/CDIP1 depletion (Fig. 5B). In some cases, cargo-containing tubules extended to the cell margin (Fig. 5B, inset).

Rab11 controls endosomal recycling pathways (Goldenring, 2015), and EHD proteins have been linked to Rab11 via interactions with Rab11FIP2 and Rab11FIP5 (Lu et al., 2015; Naslavsky et al., 2006; Solinger et al., 2020). The effect of LITAF/CDIP1 depletion on Rab11 distribution was particularly pronounced, since extensive tubules labelled with endogenous Rab11 were rarely seen in control cells but were densely populated in LITAF/CDIP1-depleted cells (Fig. 5C). Likewise, EGFR, which, when not bound to ligand, recycles via a Rab11 compartment (Baumdick et al., 2015), localised to numerous tubules in serum-starved LITAF/CDIP1-depleted cells but far fewer in control cells (Fig. S5C). EGFR tubule density increased more than 5-fold in LITAF/CDIP1-depleted cells compared to control cells (Fig. S5D).

Using the marked degree of EGFR tubulation as a reference, phenotypes were seen upon knockdown of either LITAF or CDIP1, using independent siRNA oligonucleotides (Fig. S5E), altogether suggesting that LITAF and CDIP1 have overlapping but non-redundant functions. The increase in EGFR-containing tubules was reversed by co-expressing siRNA-resistant LITAF and CDIP1, with EGFR instead distributing to LITAF/CDIP1-rich endosomes (Fig. S5F,G).

Underscoring their broad impact on recycling compartments, LITAF/CDIP1 depletion also greatly exaggerated the extent of SNX1 tubules (Fig. 5D). To further explore the consequences of depleting LITAF/CDIP1, we examined cells by EM. Linear membrane tubules, ∼100 nm in diameter, were readily apparent in LITAF/CDIP1-depleted cells but not in control cells (Fig. 5E, arrowheads). To examine whether a portion of these tubules opened to the cell exterior, we stained cells with Ruthenium Red (RR), a membrane-impermeable EM marker (Deneka et al., 2007), concomitant with cell fixation (Fig. 5F). In control cells, only the cell surface was stained. In contrast, RR frequently labelled tubules in LITAF/CDIP1-depleted cells that penetrated deep into the cytoplasm, and sometimes connected to endosome-like internal compartments (Fig. 5F, arrowheads). Hence, the absence of LITAF and CDIP1 disrupts compartmental boundaries within the endosomal system.

**Fig. 6.**
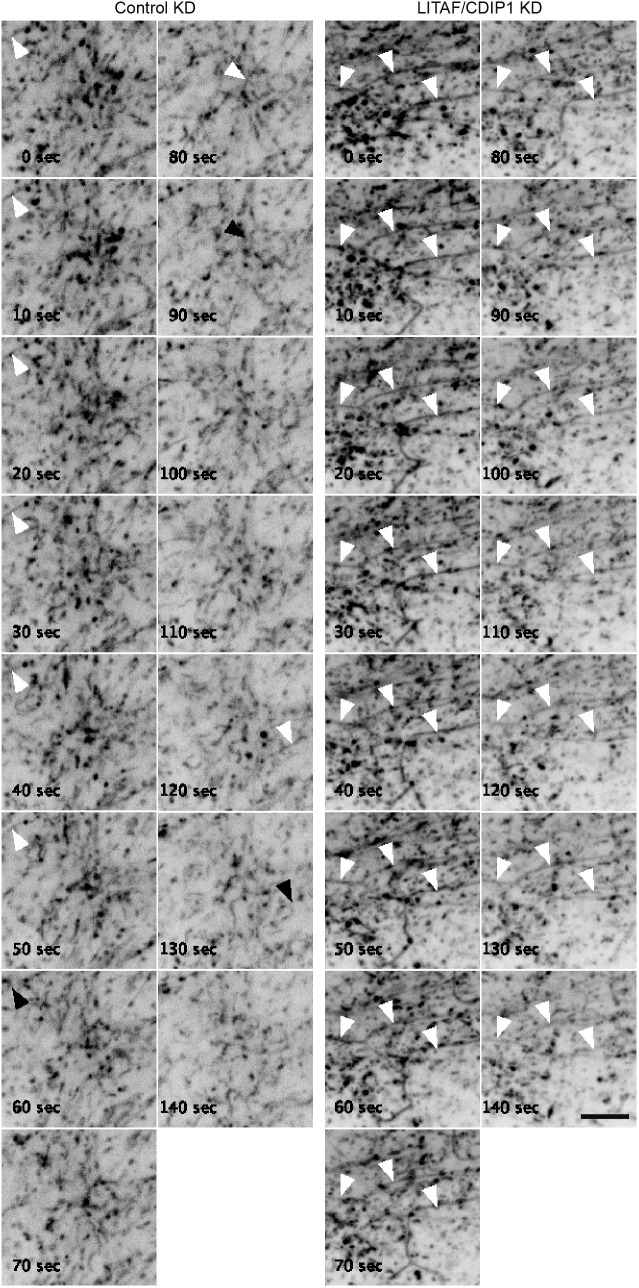
**LITAF/CDIP1 support scission of Rab11 tubules.** Control or LITAF/CDIP1-depleted HeLaM cells transfected with GFP–Rab11 and imaged by time-lapse spinning disc confocal microscopy (see also Movie 1). Frames are shown at 10 s intervals. White arrowheads point to Rab11 tubules; black arrowheads show the position of Rab11 tubules in the previous frame(s) that have now disappeared. See Movie 1. Scale bar: 5 µm.

The extensive tubulation of recycling compartments upon LITAF/CDIP1 depletion could reflect a defect in tubule scission. To further explore this, we examined the dynamic behaviour of GFP–Rab11 (this construct uses Rab11a). In control cells, GFP–Rab11 was highly dynamic, associating with motile vesicles and short-lived GFP–Rab11 tubules (Movie 1), in line with previous reports that Rab11 associates with transient tubular structures that emanate from endosomes (Campa et al., 2018; Sönnichsen et al., 2000). Stable GFP–Rab11 tubules were rare, with only 6/325 (1.8%) of GFP–Rab11 tubules >2 µm long lasting longer than 100 s. Such long-lasting GFP–Rab11 tubules were ∼10 times more frequent in LITAF/CDIP1-depleted cells (35/169; 20.7%) (Fig. 6), although high numbers of dynamic tubules were still observed (Movie 1). Hence, our data suggest that LITAF and CDIP1 limit the expansion of tubular recycling compartments by supporting dynamic membrane scission.

### LITAF and CDIP1 support integrin recycling and cell migration

Since LITAF and CDIP1 control the scission of recycling tubules, we tested whether they are important for cargo recycling. We first examined the kinetics of the transferrin receptor, which recycles both fast through a Rab4 pathway and more slowly via Rab11. In contrast to previous reports (Moriwaki et al., 2018), we detected no alteration in the rate of transferrin recycling, despite efficient depletion of LITAF and CDIP1 (Fig. S6A–C).

**Fig. 7.**
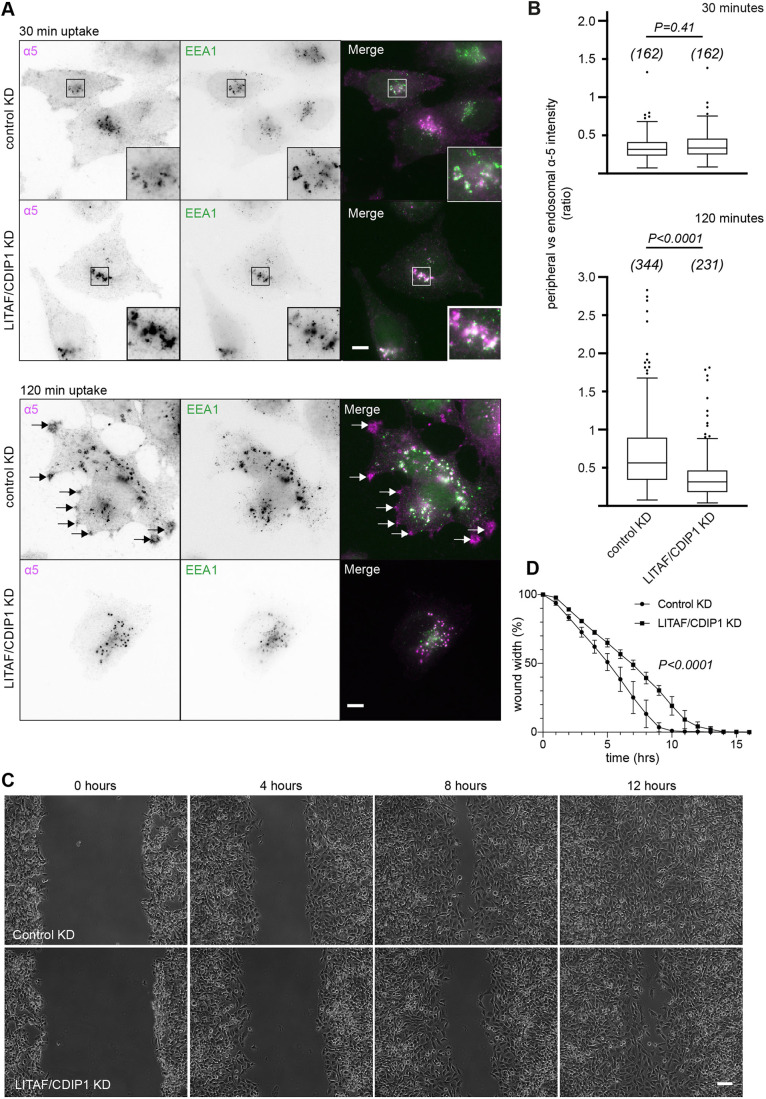
**LITAF/CDIP1 support integrin recycling and cell migration.** Control or LITAF/CDIP-depleted A2780 cells were pulse-labelled with anti-α5 integrin antibody for 30 min at 4°C, then incubated at 37°C for 30 or 120 min and fixed and stained. Representative widefield images are shown. Scale bar: 10 µm. (B) The relative intensity of anti-α5 integrin staining at the leading edge verses the internal pool at 30 min and 120 min chase was quantified using ImageJ software. Cells numbers are shown in brackets. Results represented as Tukey box plot with outliers shown. *P*-values calculated with a Kolmogorov–Smirnov test. (C) Control or LITAF/CDIP1-depleted A2780 cell monolayers were manually scratched and time-lapse imaged. Scale bar: 100 µm. (D) The wound width from C was quantified at each timepoint using ImageJ software. The percentage of the wound area remaining is shown versus time. Error bars are s.d. from triplicate experiments. Statistical analysis was by unpaired *t*-test with Welch's correction [t=4.6, d.f.=182.1].

We then examined the trafficking of α5β1 integrin, which exhibits polarised recycling that is controlled by Rab11, ARF6 (Powelka et al., 2004) and EHD1 (Jović et al., 2007; Salem et al., 2015), using a fluorescence assay based on the uptake of anti-α5 integrin antibodies. Anti-α5 integrin was internalised to label EEA1-positive endosomes in both control and LITAF/CDIP1-depleted A2780 cells (Fig. 7A). However, whereas a significant amount of internalised anti-α5 integrin antibody subsequently relocalised to polarised peripheral compartments in control cells, it remained almost exclusively in endosomes in LITAF/CDIP1-depleted cells (Fig. 7A,B). Consistent with a defect in polarised integrin recycling, LITAF/CDIP1 depletion caused a significant reduction in A2780 cell migration as measured by wound closure assays (Fig. 7C,D).

In summary, we identify LITAF as a novel membrane curvature protein that supports the scission of recycling membrane tubules. As such, LITAF contributes to the polarised trafficking of integrins and supports cell migration.

## DISCUSSION

Here, we show that LITAF, a monotopic integral membrane protein (Ho et al., 2016; Qin et al., 2016), supports membrane curvature-dependent compartmentalisation within the endocytic pathway. Specifically, LITAF and the closely related CDIP1 orchestrate the morphogenesis of Rab11- and ARF6-dependent tubular endosomal recycling compartments by supporting membrane scission.

The C-terminal SLD of LITAF contains a proposed 22-amino-acid membrane anchor that is predicted to form an amphipathic helix (Ho et al., 2016), flanked by the knuckles of a zinc finger (Ho et al., 2016; Ponting, 2001; Qin et al., 2016). An amphipathic helix that embeds shallowly into lipid bilayers is a common feature of proteins that promote membrane curvature (Campelo et al., 2008; Kozlov et al., 2014). Many of these proteins associate with membranes reversibly, and their membrane-deforming properties are revealed by the induction of tubules from pre-formed liposomes (Ford et al., 2002). However, like reticulons, LITAF is an integral membrane protein. For reticulons, the induction of membrane curvature is manifested by a reduction in the size of liposomes generated from lipid–protein–detergent mixes (Hu et al., 2008; Khaminets et al., 2015), and this is also seen for LITAF. These data, alongside the finding that some cell lines display Pacsin2 tubules only when the LITAF SLD is present, indicate that LITAF may induce, as well as support, membrane curvature. Further mechanistic studies should clarify this issue.

We found that the membrane-integrated region of LITAF contributes towards membrane curvature because conserved cysteine residues within the membrane anchor confer partitioning into high-curvature recycling tubules. However, as yet we do not know whether the amphipathic nature of this region is important for this activity. Other features of the SLD are likely to be important for its tubular partitioning. Notably, the SLD zinc finger is essential for membrane integration (Ho et al., 2016; Qin et al., 2016). We speculate that it not only stabilises the membrane region in the membrane but might also clamp it so as to ensure that it remains shallowly embedded in the membrane and thus capable of inducing or supporting curvature. Many membrane curvature proteins oligomerise into membrane-associated scaffolds (Kozlov et al., 2014; Shibata et al., 2009; Simunovic et al., 2018). LITAF also self-associates in a manner dependent on the SLD domain (Lee et al., 2012). Here, we show LITAF also associates with two TRE proteins, Pacsin2 and EHD1, and that these interactions may allow LITAF oligomers to concentrate at regions of TRE scission. Finally, like EHD1 (Jović et al., 2009), LITAF binds to PtdIns4P (Moriwaki et al., 2018), a lipid implicated in recycling, which may also act to concentrate LITAF close to the point of membrane scission. Hence, there are several pathways that could contribute to the membrane curvature-inducing activity of LITAF.

The association of LITAF with EHD1, and the similar phenotypes seen when depleting LITAF/CDIP1 to that seen when depleting EHD1 (Cai et al., 2013), place LITAF function alongside that of this scission ATPase. EHD1 is implicated in the scission of Rab11-containing recycling structures (Solinger et al., 2020), in line with the marked stabilisation of a subset of Rab11 tubules upon LITAF/CDIP1 depletion. EHD1 is a mechanical ATPase (Daumke et al., 2007; Deo et al., 2018), and thus would provide the driving force for membrane scission. LITAF could support scission by helping to organise EHD1 at the membrane and/or by enhancing the degree of local membrane distortion imposed by EHD1 ATPase activity. Notably, the LITAF SLD partitions into tubules with a uniform diameter of ∼55 nm, which is considerably narrower than the 100 nm tubules decorated by overexpressed EHD1 (Caplan et al., 2002). Finally, it is also possible that LITAF supports membrane scission by regulating WASH- and actin-dependent membrane domains, and/or controlling motor activities that contribute to membrane scission (Cullen and Steinberg, 2018; Etoh and Fukuda, 2019).

Our data suggest that LITAF is not obligate for all recycling pathways, since we found that LITAF/CDIP1 depletion does not affect bulk recycling rates as determined by transferrin recycling, in contrast to a previous report (Moriwaki et al., 2018). However, LITAF supports the appropriate morphogenesis of recycling tubules containing SNX1, as well as Rab11 and TRE components. Hence, LITAF might play a general role in several parallel recycling pathways that involve membrane tubulation. Alternatively, like the recently identified FERARI complex (Solinger et al., 2020), LITAF may act as a molecular scaffold between EHD1, Rab11 and SNX1, and thus coordinate cargo movement through a series of functionally linked recycling intermediates. Aside from the polarised recycling of integrins, we do not yet know the range of recycling cargoes that depend on LITAF. One possibility is that LITAF, which binds to Nedd4 family ubiquitin ligases (Eaton et al., 2011; Shirk et al., 2005) and is linked to ubiquitin homeostasis (Li et al., 2015), may act on cargo whose endocytic fate is controlled by ubiquitylation. In this context, the regulation of integrin trafficking by ubiquitylation (Kharitidi et al., 2015) may be significant.

The morphological consequences of disrupting LITAF function may be cell type dependent. Hence, in HeLa and other cell types in which the tubular morphology of recycling compartments is already relatively pronounced, the delay in membrane scission caused by depletion of LITAF results in very extensive tubules, with some opening to the cell exterior. Such loss of compartmentalisation could have severe long-term cellular consequences. In other cells, such as A2780 cells, reduction in membrane scission appears to manifest simply as a slowing of cargo exit from the endosome. Impaired recycling would also lead to the build-up of membrane within the endolysosomal system and thus could contribute towards endosomal vacuolation, a further morphological manifestation of impaired LITAF function (Edgar et al., 2020; Lee et al., 2012; Zhu et al., 2013).

The role of LITAF in maintaining membrane organisation during endosomal recycling is likely to be important for understanding CMT disease, and is consistent with other studies linking demyelinating forms of CMT to mutations in endocytic regulators that impair exit from the early endosome (Azzedine et al., 2012; DiVincenzo et al., 2014). Of note, myotubularins support the hydrolysis of PtdIns3P to promote exit from the endosome (Wallroth and Haucke, 2018), and MTMR2, MTMR5 and MTMR13 are mutated in CMT4B1– CMT4B3, respectively (Azzedine et al., 2003; Bolino et al., 2000; Nakhro et al., 2013; Senderek et al., 2003). Fig4, a component of the PIKfyve complex required for generating the late endosomal lipid PtdIns3,5P_2_, is mutated in individuals with CMT4J (Chow et al., 2007), which shares morphological similarities to CMT1C (Edgar et al., 2020). Furthermore, similar to our finding that CMT1C disease mutations impair the partitioning of LITAF into recycling endosomes, mutations in SH3CT2 that mislocalise this protein from the recycling endosome to the sorting endosome cause CMT4B (Roberts et al., 2010). Since the development and maintenance of myelin involves Schwann cell migration and the polarised recycling of myelin proteins, subtle impairment of recycling caused by CMT mutations in LITAF may affect Schwann cells disproportionately (Pereira et al., 2012). Future work should establish the relationship between LITAF and Schwann cell factors.

## MATERIALS AND METHODS

### Antibodies and reagents

TAT1 anti-tubulin was a gift from Keith Gull, University of Oxford, UK [1:2500 for immunofluorescence (IF) (Stefani et al., 2011)]. Sheep anti-Pacsin2 was produced in-house (Billcliff et al., 2016). The following commercial antibodies were used. Mouse: anti-LITAF [Santa Cruz Biotechnology; C-5; Cat. Sc-166719; batch G0816; 1:2000 for western blotting (WB), 1:200 for IF]; anti-MHC class I (Santa Cruz Biotechnology; W6/32; Cat. Sc-32235; 1:200 for IF); anti-CD59 (Thermo Fisher Scientific; MEM43; Cat. MA-19133; batch 75162694; 1:200 for IF); anti-HA (Santa Cruz Biotechnology; F-7; Cat. Sc-7392; batch K1918; 1:200 for IF); anti-Strep (Novagen; Cat. 71590-3; batch D00167046; 1:1000 for WB, 1:200 for IF); anti-CD63 (Millipore; RFAC4; Cat. CBL553; batch 3153282); anti-EEA1 (BD Biosciences; 14/EEA1; Cat. 610457; batch 6302827; 1:400 for IF); anti-MICAL-L1 (Abnova; B01P; Cat. H00085377-B01P; batch FC071; 1:200 for IF); anti-SNX1 (BD Biosciences; 51/SNX1; Cat. 611482; batch 8179905; 1: 400 for IF); anti-CD98 (Biolegend; MEM-108; Cat. 315602; batch B223166; 1:200 for IF); anti-PI4P (Echelon; Cat. Z-P004; batch AB092517-21; 1:200 for IF) (Hammond et al., 2009); anti-α5 integrin (Invitrogen; eBioSAM-1; Cat. 14-0496-82; batch 21020; 1:200 for IF); anti-EGFR [ATCC; MAb 108, purified from the hybridoma cell line HB-9764; 1:500 for IF (Stefani et al., 2011)]. Rabbit: anti-Rab11 (Proteintech; Cat. 15903-1-AP; 1:200 for IF); anti-EEA1 (Cell Signaling Technologies; C45B10; Cat. 32885; 1:400 for IF); anti-CDIP1 [Peptide Speciality Laboratories; 1:1000 for WB (Qin et al., 2016)]; anti-V5 (Abcam; Cat. Ab9116; batch GR256657-1; 1:1000 for WB, 1:200 for IF); anti-GFP (Abcam; Cat. Ab290; batch GR3321614-1; 1:200 for IF). Sheep: anti-TGN46 (Bio-Rad; Cat. AHP500; 1:200 for IF). Goat: anti-Myc tag (Abcam; Cat. Ab9132; batch GR215199-3; 1:1000 for WB, 1:200 for IF). Fluorescent secondary antibodies were from Jackson ImmunoResearch Laboratories (PA, USA): Alexa Fluor 680-conjugated AffiniPure donkey anti-rabbit IgG (Cat. 711-625-152; batch 139907; 1:5000 for WB); Alexa Fluor 680-conjugated AffiniPure Donkey Anti-Mouse IgG (Cat. 715-625-150; batch 145146/153931; 1:5000 for WB; Alexa Fluor 790-conjugated AffiniPure donkey anti-rabbit IgG (Cat. 711-655-152; batch 132235; 1:5000 for WB); Alexa Fluor 790-conjugated AffiniPure donkey anti-goat IgG (Cat. 705-655-147; batch 143129; 1:5000 for WB); Alexa Fluor 680-conjugated AffiniPure donkey anti-rabbit IgG (Cat. 711-625-152; batch 149402; 1:5000 for WB); Alexa Fluor 680-conjugated AffiniPure donkey anti-goat IgG (Cat. 705-625-147; batch 133233; 1:5000 for WB); Alexa Fluor 594-conjugated AffiniPure donkey anti-mouse IgM (Cat. 715-585-020; batch 134334; 1:800 for IF); Alexa Fluor 488-conjugated AffiniPure donkey anti-mouse IgG (Cat. 715-545-151; 1:800 for IF); Alexa Fluor 594-conjugated AffiniPure donkey anti-mouse IgG (Cat. 715-585-151; 1:800 for IF); Alexa Fluor 488-conjugated AffiniPure donkey anti-rabbit IgG (Cat. 711-545-152; 1:800 for IF); Alexa Fluor^®^ 594-conjugated AffiniPure donkey anti-rabbit IgG (Cat. 711-585-152; 1:800 for IF). GFP-P4M was from Addgene (#51469).

### Cell culture and transfection

HeLaM cells (a gift from Margaret Robinson, University of Cambridge, UK), HEK293T and Cos7 cells (ATCC) were grown in Dulbecco's modified Eagle's medium (DMEM). Vero cells were grown in Eagle's minimum essential medium (EMEM), and U20S cells in McCoy's 5a medium. A2780 cells were grown in RPMI. Cell lines were checked routinely for contamination. All media were supplemented with 1% non-essential amino acids (NEAA) and 10% fetal calf serum (HyClone; Perbio). Transient transfections were performed using JetPei (Polyplus) or Fugene6 (Roche). Transfection with siRNA was performed using Interferin (Polyplus). All siRNAs were ON-TARGETplus from Dharmacon. The LITAF siRNA oligo sequences were 08: 5′-UGCAGGACGUGGACCAUUA-3′; 05: 5′-GCAUGAAUCCUCCUUGGUA-3′; CDIP1 siRNA sequences were 17: 5′-GGACAACACCAACACGUAA-3′; 18: 5′-CGUACAAGCGCCUGUGCUA-3′; 19: CCUACAUGCCUCCGGGUUU-3′; 20: ACUUUCAAGGAUGUGACGCA-3′. For most experiments oligonucleotide 5 (LITAF) and 20 (CDIP1) were used. Allstars non-targeting siRNA (Qiagen) was used as a control. Knockdown efficiency was assessed by western blotting. To induce TGN tubules, cells were incubated 5 µg/ml Brefeldin A for 1 h.

### Fluorescence microscopy and trafficking experiments

Fluorescence was imaged on an Olympus BX60 upright microscope fitted with a 60×1.4 NA Plan Apo objective and CoolSnap ES camera, and 12-bit images captured using MetaVue software. For some experiments, cells were imaged using 60×1.4 NA PlanApo or 100×1.35 N.A. UPlanFl objectives on an Olympus IX70 microscope equipped for optical sectioning microscopy (Deltavision; Applied Precision, Issaquah, WA, USA) and a CoolSnap HQ camera (Photometrics, Marlow, UK). Each *z*-series (0.2 μm intervals) was deconvolved and projected using SoftWorx (Applied Precision). For confocal microscopy images were acquired using a Leica TCS SP8 AOBS inverted confocal using a 100×/1.4 PL APO objective. Only the maximum intensity projections of selected 3D stacks are shown in the results. All images were opened as 16-bit greyscale images and scaled using linear transformations in ImageJ, then converted into 24-bit RGB files in PhotoShop CS (Adobe). Regions of interest in each figure panel are magnified by 3-fold. Files were placed in Illustrator CS (Adobe).

For live-cell imaging of Rab11, images were acquired using a CSU-X1 spinning disc confocal (Yokagowa) on a Zeiss Axio-Observer Z1 microscope with a 60×/1.40 NA Plan-Apochromat objective, Evolve EMCCD camera (Photometrics) and motorised *XYZ* stage (ASI). The 488 nm and 561 nm lasers were controlled using an AOTF through the laser stack [Intelligent Imaging Innovations (3I)] allowing both rapid ‘shuttering’ of the laser and attenuation of the laser power. Slidebook 6 software (3i: Intelligent Imaging Innovations, Inc.) was used to capture images every 5 s. Movies were analysed with ImageJ software.

The relative cellular density of TRE tubules was estimated from fluorescence images using a non-biased, morphometric technique. First, random views of cells were imaged and a grid was imposed using ImageJ. Grid intersections falling inside of cells were counted to provide an estimate of total relative cell area. The frequency that tubule profiles crossed the horizontal lines of the grid was also counted, to provide an estimate of the total relative tubule length within each cell. The ratio of tubule count to cell count provided an estimate of the relative volume density of tubules in each cell. Values were obtained from multiple images from at least three independent experiments, and statistical analysis was performed using Prism 9 (GraphPad Sofware, LLC). To quantify the colocalisation of tubule markers, a grid was superimposed on each image and each tubule that crossed a horizontal grid line was examined for colabelling of markers.

### Trafficking experiments

For MHC class I, CD59 and CD98 pulse-labelling experiments, HeLaM cells grown on glass coverslips were serum starved for a minimum of 2 h then incubated with MHC class I, CD59 or CD98 antibodies for indicated times at 37°C in binding medium [Leibovitz's L-15 medium containing 0.2% (w/v) BSA], then acid stripped (0.5% acetic acid, 500 mM NaCl pH 3.0) for 1 min at 37°C to remove the cell surface pool (Reineke et al., 2015). Cells were then fixed in 3.7% PFA, quenched in glycine and incubated with secondary antibodies and DAPI in 0.2% saponin for 30 min. Integrin trafficking was performed using A2780 cells which had been starved for 15 min, then incubated with anti-α5 antibody (1:200). Cells were fixed in 3.7% PFA and permeabilised with 0.1% Triton X-100 for 5 min before antibody labelling. For transferrin recycling, HeLaM cells were incubated with 5 µg/ml biotin-XX-transferrin (Thermo Fisher Scientific) for 1 h at 37°C. Cells were then washed in cold PBS containing calcium and magnesium (Sigma) and incubated for 10 min in wash buffer [150 mM NaCl, 2 mM CaCl_2_, 20 mM NaOAc pH5.0 and 50 μg/ml desferroxamine (Sigma)], and a second incubation in PBS containing 50 μg/ml desferroxamine. Cells were chased at 37°C for the indicated time points in media containing 200 μg/ml unlabelled transferrin (Sigma) and 50 μg/ml desferroxamine. At each timepoint cells were washed with PBS and lysed in SDS-PAGE sample buffer for western blot analysis.

### Cell migration assay

Control or LITAF and CDIP double-knockdown A2780 cells were plated into Ibidi glass-bottomed plates. Immediately prior to imaging the cell monolayer was manually scratched to create a gap. Images were acquired on an Eclipse Ti inverted microscope (Nikon) using a 10×/0.45 NA SPlan Fluar objective using NIS Elements AR.46.00.0. imaging software. Point visiting was used to allow multiple positions to be imaged within the same time-course and cells were maintained at 37°C and 5% CO_2_. The images were collected every hour for 24 h using a Retiga R6 (Q-Imaging) camera. The data were analysed using ImageJ software to determine the gap width at each time point.

### Molecular reagents and recombinant protein

The following constructs were generated by standard cloning techniques: Strep-, GFP-, Myc-, His_6_-, V5- and RFP-tagged LITAF and LITAF(SLD), GFP–CDIP, GFP–RAB11, HA–ARF6, GFP–EHD1 and GFP–Pacsin2. Standard site-directed mutagenesis was used to generate point mutations. His_6_–Sec61β-OPG in pHisTrx was a gift from Stephen High, University of Manchester, UK. His-tagged proteins were expressed in *Escherichia coli* strain BL21 (DE3) pLysS by IPTG induction. Cell pellets were resuspended in 50 mM Tris-HCl pH 7.4, 300 mM NaCl and 10 mM imidazole, supplemented with 1 mM PMSF, complete protease inhibitor cocktail (Roche), Benzonase, and 0.5% lauryldimethylamine-N-Oxide (LDAO). The cells were lysed by sonication and insoluble material was pelleted at 17,000 ***g*** for 60 min at 4°C. His-tagged LITAF was isolated using a 1 ml Ni-NTA HisTrap column connected to an ÄKTA purifier FPLC (GE Healthcare), and eluted using an imidazole gradient. A further purification step by size exclusion chromatography using a Superdex 200 increase column (GE Healthcare) in 50 mM Tris-HCl pH 7.4,, 300 mM NaCl, and 0.5% LDAO was included.

### Electron microscopy

For liposome reconstitution experiments, liposomes were first prepared by dissolving 7:3 (molecular ratio) of phosphocholine (PC) and cholesterol in chloroform, followed by drying under argon and resuspending to 10 mg/ml in 50 mM NaCl, 10 mM Tris-HCl pH 7.5. Liposomes were diluted to 0.1 mg/ml in 100 µl mixture containing 0.3 mg/ml detergent-solubilised protein (or an equal volume of buffer with detergent as negative control). These mixtures were added to fresh tubes containing Bio-Beads™ (Bio-Rad; 100 µl of mixture per 50 mg of Bio-Beads™, pre-washed in 150 mM NaCl, 10 mM Tris-HCl, pH 8.0 with overnight rotation) to remove the detergent. The tubes were inverted and vibrated on a shaker-mixer for 1 h, pelleted, and the supernatant transferred to another tube with fresh Bio-Beads™. This process was repeated three times. A 3 µl sample of each mixture was dropped on a carbon-coated EM grid and left for 5 min. The liquid was carefully removed by touching the grid onto filter paper, and the grid was left to dry. The dried grid was floated briefly on a drop of 2% uranyl acetate and then removed. The grid was dried once more as above and incubated for a further 15 s with 2% uranyl acetate before drying again. The grid was then washed twice in deionized water as above, and then examined by EM. Samples were examined using a FEI BioTWIN microscope. For size analysis, the diameters of liposomes from randomly selected images were examined. Where liposomes were occasionally ellipsoid, the longest diameter was determined.

For routine cellular EM, cells were fixed in cacodylate buffer containing a formaldehyde and glutaraldehyde mix, then sequentially treated with reduced OsO_4_ for 1.5 h, 1% tannic acid for 1 h, and 2% UO_2_(CH_3_COO^−^)_2_ overnight. The stained cells were dehydrated, and then embedded in resin before viewing on a FEI BioTWIN microscope. For Ruthenium Red staining, live cells were first treated with 1.33% glutaraldehyde and 0.33% Ruthenium Red (freshly made from Ruthenium Red powder) in 0.067 M cacodylate buffer at 37°C for 1 h, then this solution was washed using H_2_O and treated by 1.33% OsO_4_ and 0.33% Ruthenium Red in 0.067 M cacodylate buffer for further 30 min. The cells were washed and scrapped off from dish for dehydration and embedding for EM.

For CLEM experiments, cells were grown on glass bottomed dishes and fixed, and optically sectioned images were acquired by DeltaVision microscopy. The locations of the cells that had been imaged were recorded using the grid as a reference. Cells were carefully embedded in resin and processed for EM. Fluorescence images were correlated to the EM images using either Reconstruct software (Version 1.1.0.0, John C. Fiala), or ec-CLEM plugin for Icy (Quantitative Image Analysis Unit at Institut Pasteur).

### Co-immunoprecipitation

For co-immunoprecipitation of LITAF with CDIP1, Pacsin2 or EHD1 HEK293T cells were transiently transfected using PEI (Sigma). Cell lysates were prepared in 10 mM Tris-HCl pH 7.4, 140 mM NaCl, 0.1% Triton X-100, containing 1 mM PMSF and protease inhibitor cocktail (Sigma). A post nuclear supernatant was obtained by centrifugation at 14,000 ***g*** for 10 min at 4°C and a sample of this was retained for analysis before the remaining supernatant was incubated overnight with GFP-Trap resin (Chromotek). The resin was then washed three times with lysis buffer and heated in SDS-PAGE sample buffer at 95°C before loading onto SDS-PAGE gels for western blotting.

### Western blotting

Samples were run on Tris-glycine SDS-PAGE gels and transferred to PVDF membranes (Millipore). Membranes were incubated overnight with primary antibodies, and with secondary antibodies for 1 h, both in Tris-buffered saline with 0.1% Tween-20 (used for washing), containing Casein as a blocking agent (Sigma). Images were acquired using a LI-COR Odyssey Fc instrument and Image Studio software.

### Statistical analysis

All analysis was performed using Prism 9 software (GraphPad Software, LLC). Histograms were plotted using box-and-whisker plots by the Tukey method. Sample numbers are indicated in brackets or by individual points. Nonparametric analysis of liposome size, tubulation and integrin recycling employed the Kolmogorov–Smirnov test. A one-way ANOVA test was employed for the multiple comparisons of CD98 tubules in Fig. 4. For wound healing assays, plots from 1–9 h (at which time the wound was virtually closed in control cells) were fitted by simple linear regression, and control versus LITAF/CDIP1 knockdown compared by two-tailed *t*-test with Welch's correction.

## Supplementary Material

Supplementary information

Reviewer comments
