# Supplementary material for: Endosomal recycling tubule scission and integrin recycling involve the membrane curvature-supporting protein LITAF
Source: J Cell Sci. 2021 Aug 3;134(15):jcs258549. doi: 10.1242/jcs.258549 (PMC8353527; doi:10.1242/jcs.258549)
Supplement: Supplementary information [file joces-134-258549-s1.pdf]

Wunderley et al. Figure 1

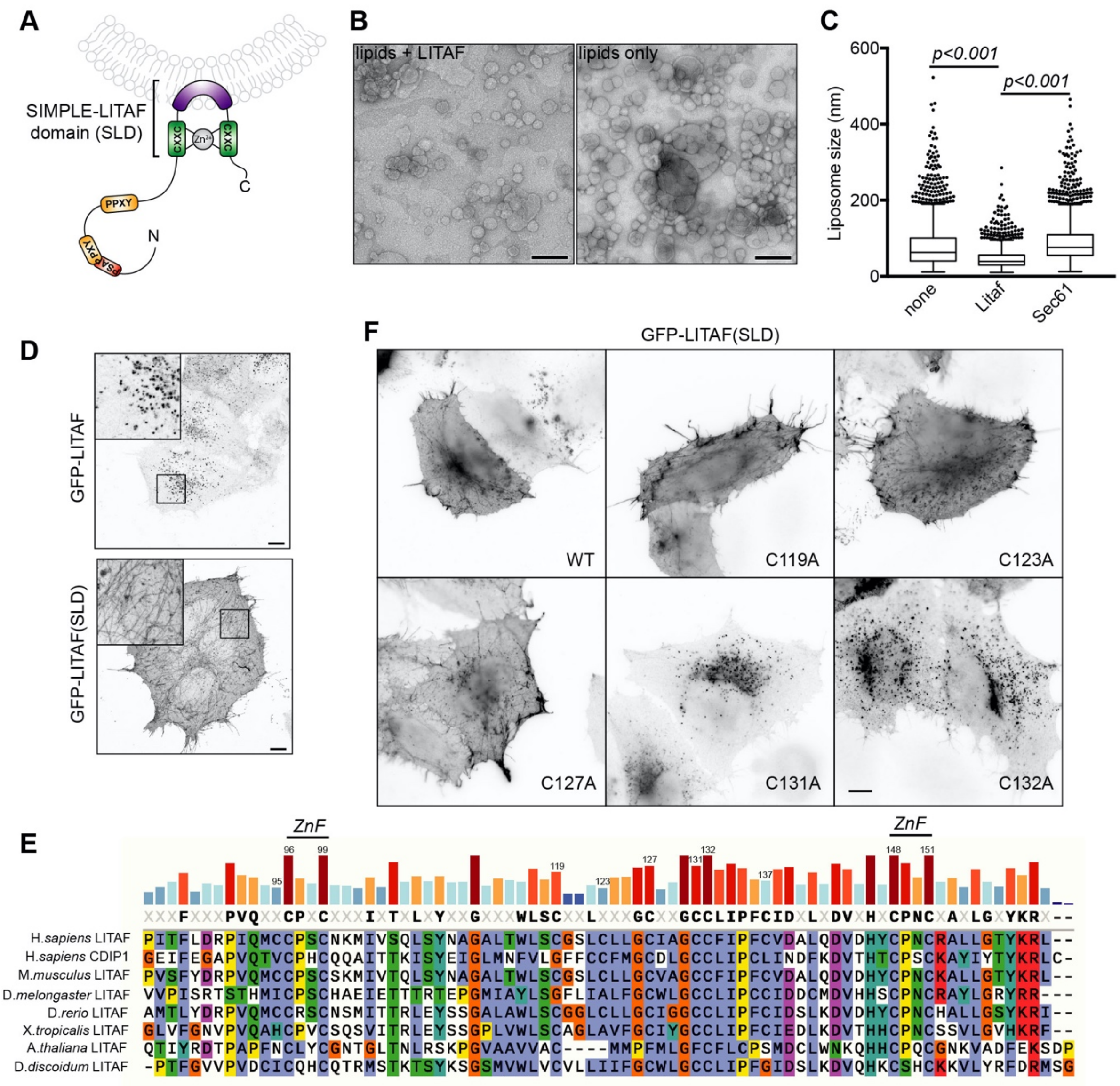

Fig. S1. (A) HeLaM cells transfected as indicated. The proportion of cells in which the construct clearly partitioned into tubules is shown. Values are means +/- SD of 3 independent experiments, 100 cells in each. (B) HeLaM cells transfected with StrepTag-LITAF or StrepTag-LITAF(SLD). Scale bar = 10 µm. (C) HeLaM cells transfected with GFP-CDIP1 or GFP-CDIP1(SLD). (D) HeLaM cells stained with anti-LITAF antibody. Confocal microscopy. (E) HeLaM cells transfected with the indicated mutants of GFP-LITAF(SLD). (F) HeLaM cells transfected with WT GFP-LITAF(SLD) or the indicated mutants and stained for CD63. Scale bars = 10 µm.

Journal of Cell Science • Supplementary information

Wunderley et al. Figure S2

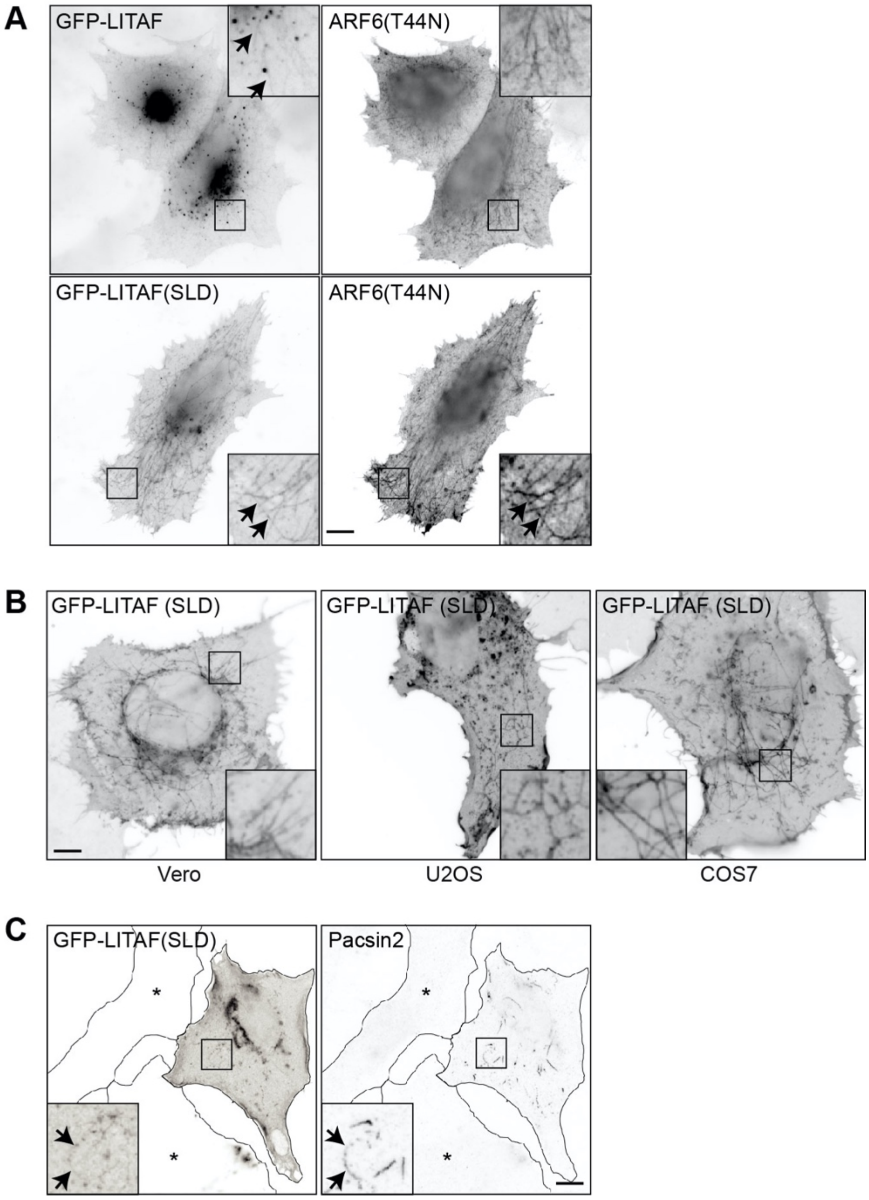

Fig. S2. (A) HeLaM cells co-transfected with GFP-LITAF or GFP-LITAF(SLD), together with HA-ARF6^{T44N}. (B) Vero, U2OS or COS7 cells transfected with GFP-LITAF(SLD). (C) Untransfected Vero cells, or cells transfected with GFP-LITAF(SLD), were stained with anti-Pacsin2. Scale bars = 10 µm.

Journal of Cell Science • Supplementary information

Wunderley et al. Figure S3

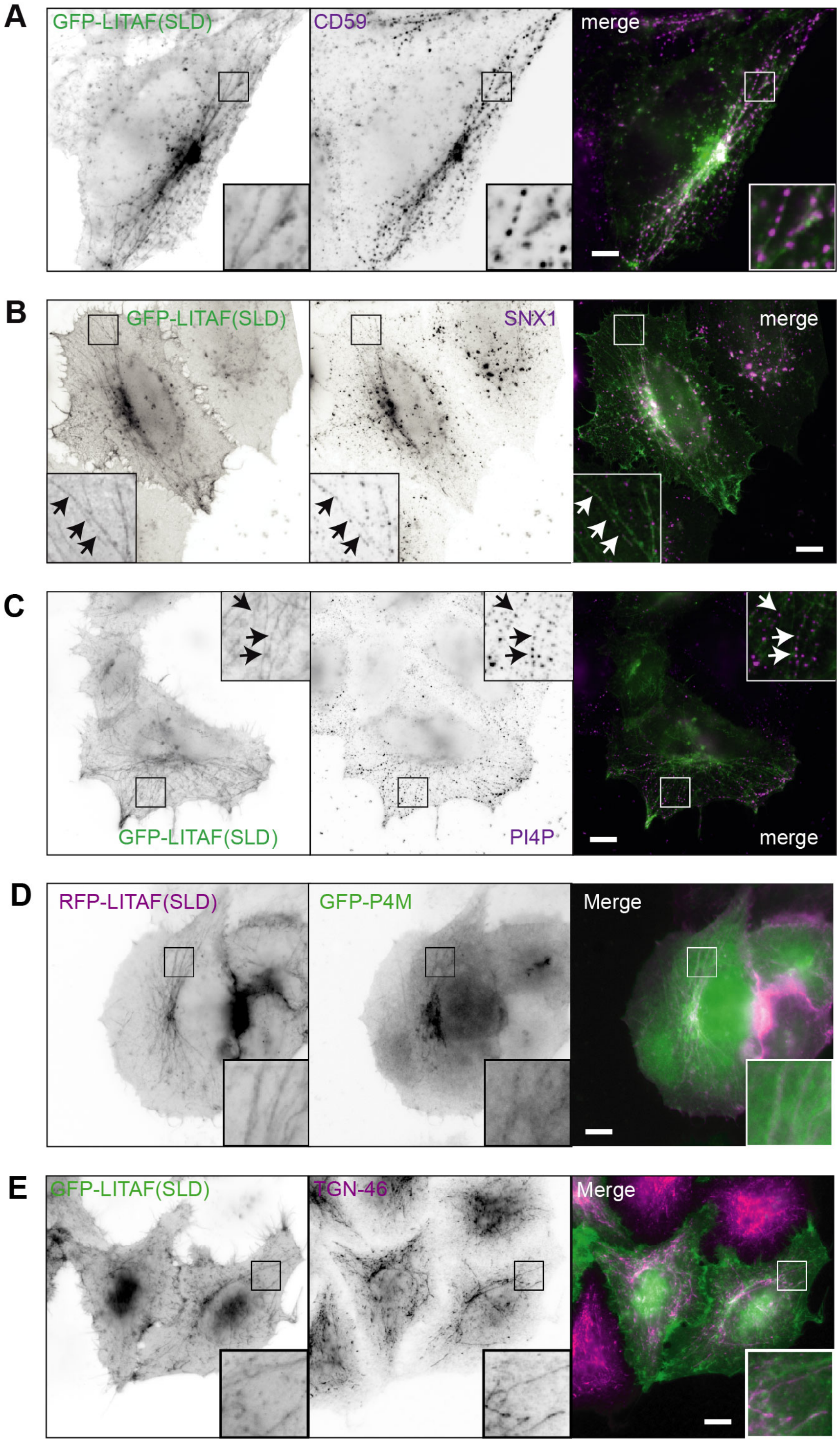

Fig. S3. (A) HeLaM cells transfected with GFP-LITAF(SLD) and pulse-labelled for 30 min with α-CD59 antibody. (B) HeLaM cells transfected with GFP-LITAF(SLD) and stained with anti-SNX1. (C) HeLaM cells transfected with GFP-LITAF(SLD) and stained with anti- PtdIns4P. (D) HeLaM cells co-transfected with RFP-LITAF(SLD) and GFP-P4M. (E) HeLaM cells transfected with GFP-LITAF(SLD) and treated with Brefeldin A were stained for TGN46. Scale bars = 10 µm.

Wunderley et al. Figure S4

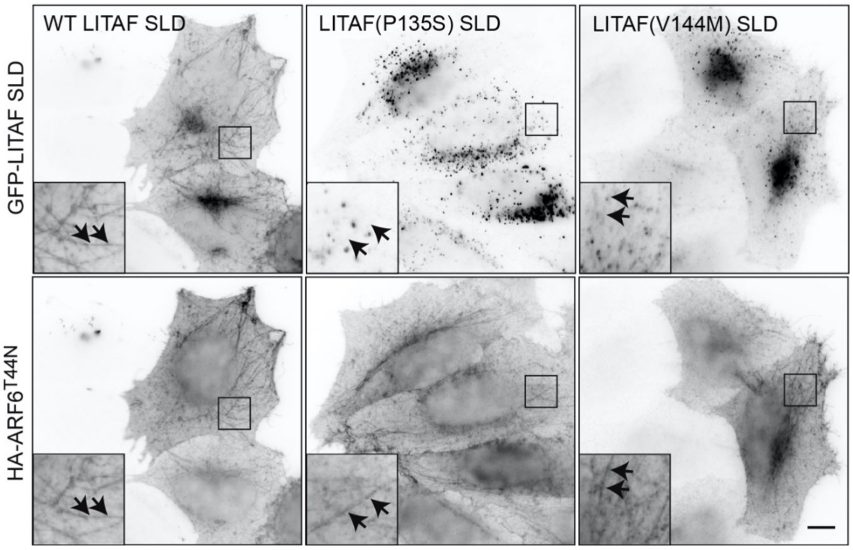

Fig. S4. HeLaM cells co-transfected with HA-ARF6^{T44N} and WT or the indicated mutants of GFP-LITAF(SLD). Scale bar = 10 μm.

Journal of Cell Science • Supplementary information

Wunderley et al. Figure S5

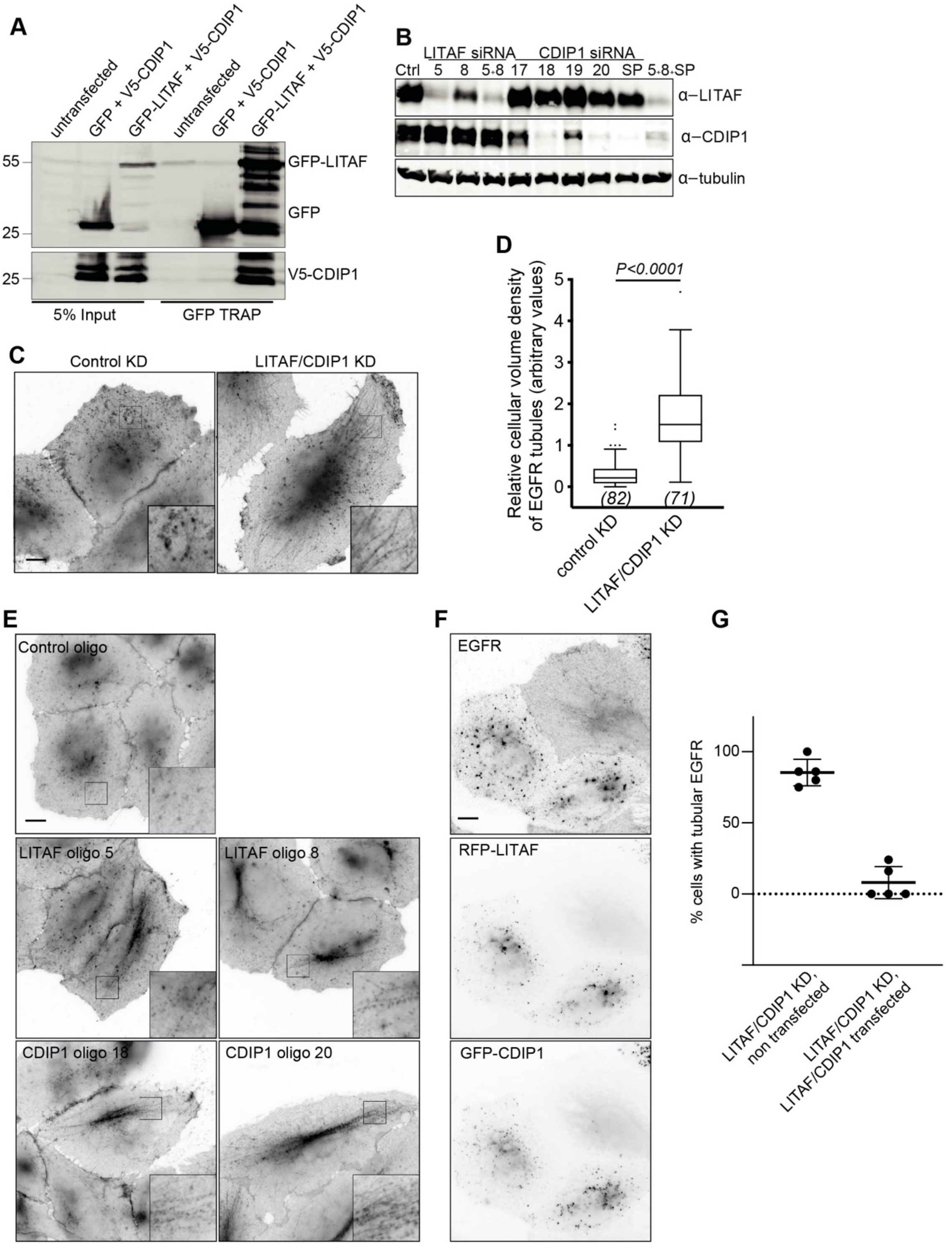

Journal of Cell Science • Supplementary information

Fig. S5. (A) HEK293 cells were co-transfected with GFP or GFP-LITAF, together with V5-CDIP1 as indicated. Cell lysates were incubated with GFP-Trap and blotted for GFP (top) or V5 (below). (B) HeLaM cells were silenced with the indicated siRNA oligos, and Western blotted. (C) Control HeLaM cells or cells depleted of LITAF and CDIP1, serum-starved and stained for EGFR. (D) Quantitation of EGFR tubules in control or LITAF/CDIP1 depleted, serum-starved HeLaM cells. (E) HeLaM cells depleted using individual siRNA oligos and stained for EGFR. (F) HeLaM cells were depleted for LITAF and CDIP1, then transfected with siRNA-resistant GFP-LITAF and RFP-CDIP1, then stained for EGFR. (G) Quantitation of RNAi rescue experiments. Cells were scored for the presence of a tubular EGFR morphology. Data from 5 independent experiments +/- SD. Scale bars = 10 μm.

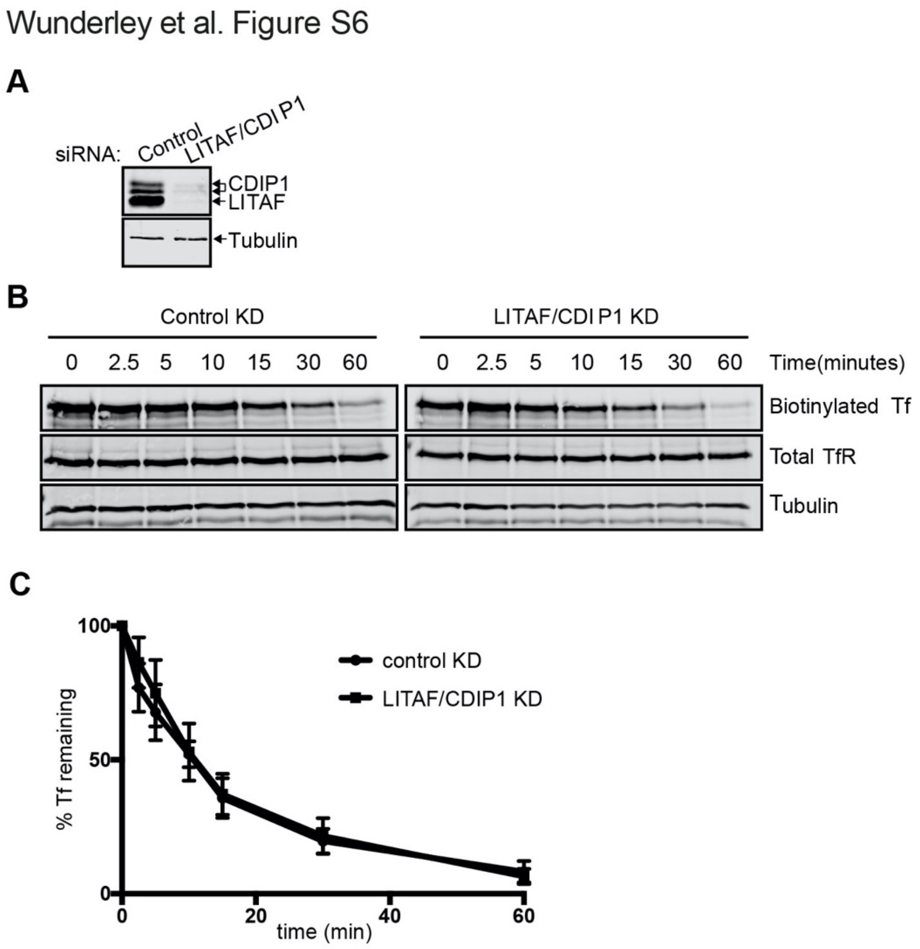

Fig. S6. (A) HeLaM cells were silenced as indicated, and Western blotted. (B) Control or LITAF/CDIP1 depleted HeLaM cells were loaded with biotinylated transferrin, chased for the indicated times in the presence of unlabelled transferrin and desferroxamine, then analysed by western blot. (C) Quantitation of transferrin recycling experiments. Values are means from 6 experiments +/- SD.

Journal of Cell Science • Supplementary information

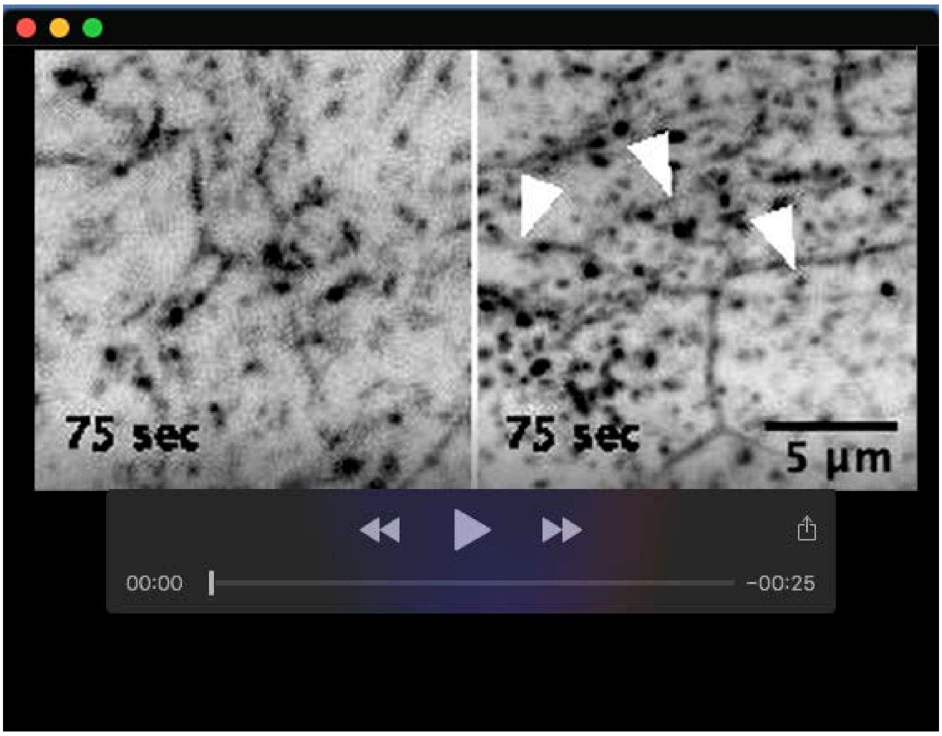

Movie 1. Movies were generated from regions of interest after time-lapse imaging of GFP-Rab11 in control (left) or LITAF/CDIP1 depleted cells (right). Original time-lapse rate is 1 frame/5 secs. White arrowheads indicate tubules > 2µm. Black arrowheads appear in the next frame after a tubule disappears.
