# Supplementary material for: Endosomal recycling tubule scission and integrin recycling involve the membrane curvature-supporting protein LITAF
Source: J Cell Sci. 2021 Aug 3;134(15):jcs258549. doi: 10.1242/jcs.258549 (PMC8353527; doi:10.1242/jcs.258549)
Supplement: Reviewer comments [file joces-134-258549_review_history.pdf]

Journal of Cell Science

Endosomal recycling tubule scission and integrin recycling involve the membrane curvature-supporting protein LITAF

Lydia Wunderley, Ling Zhang, Rebecca Yarwood, Wenxia Qin, Martin Lowe and Philip Woodman
DOI: 10.1242/jcs.258549

Editor: Giampietro Schiavo

Review timeline

Original submission:	15 February 2021
Editorial decision:	15 March 2021
First revision received:	11 June 2021
Accepted:	16 June 2021

Original submission

<u>First decision letter</u>

MS ID#: JOCES/2021/258549

MS TITLE: Endosomal recycling tubule scission and integrin recycling is facilitated by the membrane curvature protein LITAF

AUTHORS: Lydia Wunderley, Ling Zhang, Rebecca Yarwood, Wenxia Qin, Martin Lowe, and Philip Woodman

ARTICLE TYPE: Research Article

We have now reached a decision on the above manuscript.

To see the reviewers' reports and a copy of this decision letter, please go to: https://submit-jcs.biologists.org and click on the 'Manuscripts with Decisions' queue in the Author Area. (Corresponding author only has access to reviews.)

As you will see, the reviewers gave supporting reports but raised some critical points that will require amendments to your manuscript. I hope that you will be able to carry these out, because I would like to be able to reconsider your paper for publication in JCS as soon as possible

We are aware that you may be experiencing disruption to the normal running of your lab that makes experimental revisions challenging. If it would be helpful, we encourage you to contact us to discuss your revision in greater detail. Please send us a point-by-point response indicating where you are able to address concerns raised (either experimentally or by changes to the text) and where you will not be able to do so within the normal timeframe of a revision. We will then provide further guidance. Please also note that we are happy to extend revision timeframes as necessary.

Please ensure that you clearly highlight all changes made in the revised manuscript. Please avoid using 'Tracked changes' in Word files as these are lost in PDF conversion.

I should be grateful if you would also provide a point-by-point response detailing how you have dealt with the points raised by the reviewers in the 'Response to Reviewers' box. Please attend to

© 2021. Published by The Company of Biologists under the terms of the Creative Commons Attribution License (https://creativecommons.org/licenses/by/4.0/).

all of the reviewers' comments. If you do not agree with any of their criticisms or suggestions please explain clearly why this is so.

Reviewer 1

Advance summary and potential significance to field

Wunderley et al. describe a role for LITAF in the scission of recycling endosomes and show the biological importance of this event by measuring changes in integrin recycling and cell migration following depletion of LITAF. This is a really nice paper. All experiments are performed to a very high standard and the quality of the data are excellent. The field has focused a lot on the formation of recycling tubules, but we don't know much about how they are snipped up prior to delivery at the plasma membrane, and this paper provides valuable mechanistic insight into that process.

Comments for the author

I have only minor comments which are straightforward for the authors to address. The most major of which is listed first.

Fig 7 Two time points are shown in A (uncertain how many timepoints were analysed - the methods do not say) but only one time point is quantified here in 7B. These experiments need to be quantified in full. In addition, the arrows in 7A (120 min) are too small to see properly on the page.

line 101 "LITAF/SIMPLE defines a novel class..." This sounds as though this paper is the first description of this monotopic integral membrane protein, whereas the papers by Ho et al. and Qin et al 2016 are repeatedly referenced. I know what the authors mean by "novel" here but it's a bit misleading.

line 183 would help the reader to know why co-expression of ARF6(T44N) is done. It's inferred but not spelled out.

Fig 2 CLEM figure. Beautiful work. Some really small niggles: 1) Not clear to me why B is not shown in its original orientation like views A and C but is rotated 90°. 2) The "a"s should be top left. It won't obscure the inset in the EM image and will give a uniform look to the figure. 3) I think the LM and EM panels should not be both a, both b etc. Perhaps a' and a" or ai and aii.

Reviewer 2

Advance summary and potential significance to field

Here, Wunderley and colleagues examine further the function of LITAF in endosomal membrane trafficking. From observing the properties of LITAF in associating with artificial liposomes and the affects of mutants in the membrane associating SLD domain, through to functional studies of endosomal recycling tubule turnover (scission) and role in integrin, MHC-I and CD59 trafficking, the authors add to our understanding of this disease associated protein (especially through the analysis of specific CMT1C associated LITAF mutants). Overall, the study appears to well constructed and conducted and adds important new information that will be of broad interest from mechanistic membrane trafficking and the role of integrins in cell migration through to implication for CMT1C.

Comments for the author

Major points:

The term 'membrane curvature protein' lacks a degree of clarity (the manuscript title should be reconsidered). 'Support membrane curvature' may be better but this still needs to be explained in more detail. As LITAF does not appear to induce membrane tubulation it may not be considered a classical amphipathic helix containing curvature inducer (a positive control would be useful here). Is LITAF therefore a curvature sensor, with the amphiphatic helices recognizing the packing defects observed within the hydrophobic core of a highly curved membrane? If so, association of LITAF to

© 2021. Published by The Company of Biologists under the terms of the Creative Commons Attribution License (https://creativecommons.org/licenses/by/4.0/).

different diameter liposomes should reflect this sensing role. Have the authors considered these experiments?

A better explanation is needed for the experiments quantifying the reduction in liposome size upon addition of LITAF. It is not immediately apparent to me how these data can be interpreted in terms of LITAF function? Why does addition of LITAF result in lipid removal from the liposomes to reduce the size? Or is the lipid volume staying the same but the liposomes are shrinking, or is LITAF selectively vesicularising the large diameter liposomes thereby leading to a shift of the bell-shaped distribution to smaller diameters?

The association of LITAF to endosomal tubules formed through a variety of proteins (ARF6, SNX1, etc) are consistent with a curvature sensor mode. How specific is this to the endosomal compartment? If TGN tubules are induced through additional of BFA, does LITAF also partition to these?

In the biochemical identification of LITAF association with EHD1 and Pacsin2, can endogenous EHD1 and Pacsin2 be pulled down through IP of LITAF? Have the authors sort to define which regions of LITAF are required for these interactions? This would open up the opportunity to define the structure:function relationship in the context of endosomal recycling tubule scission.

Minor Comments

What is the significance of the *V5-LITAF in Fig. 3C? Is this a reflection of Ubq?

FERARI complex only has on R (line 334, page 15).

While the authors focus their interpretation of the extended endosomal tabulation observed upon LITAF KD to scission (because of binding to EHD1 and Pacsin2), it should not be completely discounted that alteration in motor coupling and/or localized WASH-dependent actin polymerization may also be significant. Perturbation in pulling and pushing forces generated by these systems also lead to formation of extended endosomal recycling tubules.

<u>Reviewer 3</u>

Advance summary and potential significance to field

This manuscript by Wunderley et al. addresses an important biological question and provides new information on the mechanisms by which endosomes undergo fission to release transport vesicles and tubules for recycling to the plasma membrane. The authors identify the LPS-Induced TNF Activating Factor known as LITAF, and a homologous protein known as CDIP1 as new participants in the process of endosomal membrane curvature supporting fission. The authors demonstrate that LITAF contains a conserved hydrophobic C-terminal region of about 22 residues known as a SIMNPLE-LITAF Domain (SLD) that harbors a zinc finger and anchors the protein to the membrane. This SLD binds PI4P and select cysteine residues were identified that allow partitioning into membrane tubules. The authors demonstrate interactions between LITAF and both Syndapin2/PACSIN2 and EHD1, known members of tubular recycling endosomes and endosomal fission machinery. KD of LITAF/CDIP1 leads to an enhanced TRE network, again suggesting impaired endosomal fission. The authors provide evidence that LITAF is required for normal recycling of select receptors, such as integrins, but not others (ie., TfR). Localizations with TRE marked by Arf6, MICAL-L1, internalized CD59 and MHC1 point to roles in recycling of clathrin-independent cargo (consistent with the role of EHD1), and indeed, similar to EHD1 depletion, the loss of LITAF/CDIP1 caused a delay in cell migration into a wound by scratch assay. Overall, this is an interesting manuscript that provides significant new information on a key and under-studied biological process, the regulation of endosomal curvature and fission.

Comments for the author

This is a well-executed and significant study that will undoubtedly contribute to our current knowledge of endosomal regulation. In general, the experiments and data are robust, convincing and nicely demonstrated. Since the manuscript covers a wide range of functions and assays, there

© 2021. Published by The Company of Biologists under the terms of the Creative Commons Attribution License (https://creativecommons.org/licenses/by/4.0/).

are several questions/points that would be helpful to address, but these critiques should not be misconstrued as negative, but rather suggestions either for this study or follow-up research.

Specific Comments/Critiques:

1) The authors might want to consider, either in the introduction or discussion, potentially addressing the role of Rab10 (and its interaction partners) in endosome biogenesis and/or fission. The recent studies from Etoh and Fukuda as well as Farmer et al. highlight the significance of Rab10 in TRE biology, and while it is understood that experimentally this is beyond the scope of the current study, addressing this and integrating it into the discussion might be helpful.

2) While this reviewer recognizes that the relationship of LITAF to PI4P-containing membranes was not a major focus of the study (it appears only in the Supplemental data), this data could be strengthened in a variety of ways, as relying exclusively on anti-lipid antibodies can be problematic, due to the inherent issues with the quality of anti-lipid antibodies.

3) Overlaying the LITAF fluorescence with a PM marker would be advisable for Fig. 1D

4) The data from Supplemental Fig. 5F should be quantified.

5) From Fig. 7, the authors infer impaired recycling of alpha5-integrins, and while that is likely the case, if possible it would be better to provide more direct evidence that measures recycling, rather than differences in subcellular distribution of the internalized protein.

6) It is recognized that this may be beyond the current (and quite wide) scope of the manuscript, but it would certainly be interesting to know whether LITAF interacts directly with EHD1, or whether it potentially interacts with Syndapin2/PACSIN2 or MICAL-L1 via proline-rich domains/SH3 domains, and that either/both of these proteins in turn interact with EHD1 through EH domain/NPF motifs.

7) Another interesting question to address is whether LITAF recruits EHD1 to endosomes for fission (again, either directly or through common interactors).

First revision

Author response to reviewers' comments

Reviewer 1 Comments for the Author:

Fig 7 Two time points are shown in A (uncertain how many timepoints were analysed - the methods do not say) but only one time point is quantified here in 7B. These experiments need to be quantified in full. In addition, the arrows in 7A (120 min) are too small to see properly on the page.

- We include quantitation of the data for both timepoints (see Figure 7B), which we agree is important to add a dynamic component to the experiment. We have also changed the arrows in the figure (Figure 7A).

line 101 "LITAF/SIMPLE defines a novel class..." This sounds as though this paper is the first description of this monotopic integral membrane protein, whereas the papers by Ho et al. and Qin et al 2016 are repeatedly referenced. I know what the authors mean by "novel" here but it's a bit misleading.

- We have altered the text accordingly (line 101: ‘novel’ omitted).

© 2021. Published by The Company of Biologists under the terms of the Creative Commons Attribution License (https://creativecommons.org/licenses/by/4.0/).

line 183 would help the reader to know why co-expression of ARF6(T44N) is done. It's inferred but not spelled out.

- We have altered the text accordingly, and also referenced the ARF6(T44N) paper correctly.

Lines 192-194

Fig 2 CLEM figure. Beautiful work. Some really small niggles: 1) Not clear to me why B is not shown in its original orientation like views A and C but is rotated 90°. 2) The "a"s should be top left. It won't obscure the inset in the EM image and will give a uniform look to the figure. 3) I think the LM and EM panels should not be both a, both b etc. Perhaps a' and a" or ai and aii.

- Corrected

Reviewer 2 Advance Summary and Potential Significance to Field:

Here, Wunderley and colleagues examine further the function of LITAF in endosomal membrane trafficking. From observing the properties of LITAF in associating with artificial liposomes and the affects of mutants in the membrane associating SLD domain, through to functional studies of endosomal recycling tubule turnover (scission) and role in integrin, MHC-I and CD59 trafficking, the authors add to our understanding of this disease associated protein (especially through the analysis of specific CMT1C associated LITAF mutants). Overall, the study appears to well constructed and conducted and adds important new information that will be of broad interest from mechanistic membrane trafficking and the role of integrins in cell migration through to implication for CMT1C.

Reviewer 2 Comments for the Author:

Major points:

The term 'membrane curvature protein' lacks a degree of clarity (the manuscript title should be reconsidered). 'Support membrane curvature' may be better but this still needs to be explained in more detail. As LITAF does not appear to induce membrane tubulation it may not be considered a classical amphipathic helix containing curvature inducer (a positive control would be useful here). Is LITAF therefore a curvature sensor, with the amphiphatic helices recognizing the packing defects observed within the hydrophobic core of a highly curved membrane? If so, association of LITAF to different diameter liposomes should reflect this sensing role. Have the authors considered these experiments?

- We have qualified the title as the reviewer suggests. We have also added some clarification to the text to address this point, accepting that our original Discussion that refers simply to an amphipathic helix may not have helped the reviewer. However, it is essential to stress that LITAF is an integral membrane protein, so experimental design and analysis that holds for amphipathic helix-containing peripheral membrane proteins such as Pacsin2 and epsin are not strictly relevant for LITAF.

We do not think that the stably integrated membrane protein LITAF supports/induces membrane curvature in precisely the same way as peripheral membrane proteins like Pacsin2 or epsin, which reversibly embed into the cytoplasmic leaflet of membranes by virtue of their amphipathic helices. We have clarified this section of the Discussion accordingly. Whilst the membrane anchor of LITAF does indeed contain a (modelled) amphipathic helix, this feature may not by itself support/induce curvature. Certainly, the membrane anchor does *contribute* to membrane curvature, since mutating cysteines within this region affects the partitioning of LITAF SLD to tubules. However, the membrane anchor is not sufficient for efficient membrane association, since mutating the Zn^{++} knuckles either side of the membrane anchor prevents membrane association (Qin et al 2016). We believe the Zn^{++} finger is not only a major driver of membrane integration, but might also be a major component supporting/inducing membrane curvature. We speculate that the Zn finger might have two roles in supporting curvature: firstly by constraining how far the membrane anchor region can embed into the membrane, secondly by orchestrating the polymerisation of LITAF into a membrane-bound scaffold. We are agnostic about whether LITAF supports vs drives membrane curvature, but now include in the Discussion some further clarification of our thinking (lines 322-

© 2021. Published by The Company of Biologists under the terms of the Creative Commons Attribution License (https://creativecommons.org/licenses/by/4.0/).

340). The LITAF SLD does appear to induce tubules *in cellulo*, since cell lines that apparently lack a tubular recycling endosome acquire this compartment when LITAF(SLD) is expressed (Fig S2), and we highlight this conclusion more explicitly (see line 166).

Since LITAF is an integral membrane protein (and hence only soluble in detergent) the size-dependent liposome binding assays are not possible. See comments below about the membrane reconstitution experiments.

A better explanation is needed for the experiments quantifying the reduction in liposome size upon addition of LITAF. It is not immediately apparent to me how these data can be interpreted in terms of LITAF function? Why does addition of LITAF result in lipid removal from the liposomes to reduce the size? Or is the lipid volume staying the same but the liposomes are shrinking, or is LITAF selectively vesicularising the large diameter liposomes thereby leading to a shift of the bell-shaped distribution to smaller diameters?

- See also previous comment. It appears we have not made the design of this experiment sufficiently clear to the reviewer. LITAF is an integral membrane protein (i.e it partitions completely with membranes and cannot be extracted with sodium carbonate, pH 11), and as such we have followed the design of experiments that test how integral membrane proteins induce membrane curvature. For peripheral membrane proteins such as epsin and Pacsin2, which are soluble in an aqueous environment, it is possible to add these proteins to prior-prepared liposomes. The induced membrane curvature is manifested by the generation of tubules. For integral membrane proteins, the situation is different, because the proteins are only soluble in the presence of detergent so cannot be added to prior-formed liposomes. For LITAF, we employed the technique designed by Hu et al. (2008) to test the curvature-inducing activity of reticulons, a well-defined example of a curvature-inducing integral membrane protein. Here, liposomes are generated from lipid-detergent-protein mixes. The end-point of such a reaction is that the curvature-inducing protein generates liposomes that are a smaller size than control liposomes; since the liposomes are being formed de novo, this is the natural way for higher curvature to be accommodated. We have amended the paragraph in the Results section (lines 110-113, 118-119) and Discussion (lines 305-319) to make this point clearer.

The association of LITAF to endosomal tubules formed through a variety of proteins (ARF6, SNX1, etc) are consistent with a curvature sensor mode. How specific is this to the endosomal compartment? If TGN tubules are induced through additional of BFA, does LITAF also partition to these?

- We have now included this nice experiment. LITAF SLD does not incorporate into BFA-induced TGN46 tubules (new panel in Figure S3). See text lines 184-187.

In the biochemical identification of LITAF association with EHD1 and Pacsin2, can endogenous EHD1 and Pacsin2 be pulled down through IP of LITAF? Have the authors sort to define which regions of LITAF are required for these interactions? This would open up the opportunity to define the structure:function relationship in the context of endosomal recycling tubule scission.

- We have attempted experiments with endogenous EHD1 and Pacsin2 but unfortunately without success. The antibodies available to us are not especially sensitive, so we are not able to pick up this transient interaction. As the reviewer suggests, defining which regions of LITAF are required for these interactions opens up a future study, but we feel this is not pertinent to the current study.

Minor Comments

*What is the significance of the *V5-LITAF in Fig. 3C? Is this a reflection of Ubq?*

- We do not yet know. It is possible that this higher form represents ubiquitination, but it is possible that another modification is in play. We have referenced this form of V5-LITAF in the figure legend.

FERARI complex only has on R (line 334, page 15).

© 2021. Published by The Company of Biologists under the terms of the Creative Commons Attribution License (https://creativecommons.org/licenses/by/4.0/).

- Corrected.

While the authors focus their interpretation of the extended endosomal tabulation observed upon LITAF KD to scission (because of binding to EHD1 and Pacsin2), it should not be completely discounted that alteration in motor coupling and/or localized WASH-dependent actin polymerization may also be significant. Perturbation in pulling and pushing forces generated by these systems also lead to formation of extended endosomal recycling tubules.

- We have noted this important idea in the Discussion (lines 352-355).

Reviewer 3 Advance Summary and Potential Significance to Field:

This manuscript by Wunderley et al. addresses an important biological question and provides new information on the mechanisms by which endosomes undergo fission to release transport vesicles and tubules for recycling to the plasma membrane. The authors identify the LPS-Induced TNF Activating Factor known as LITAF, and a homologous protein known as CDIP1 as new participants in the process of endosomal membrane curvature supporting fission. The authors demonstrate that LITAF contains a conserved hydrophobic C-terminal region of about 22 residues known as a SIMNPLE-LITAF Domain (SLD) that harbors a zinc finger and anchors the protein to the membrane. This SLD binds PI4P and select cysteine residues were identified that allow partitioning into membrane tubules. The authors demonstrate interactions between LITAF and both Syndapin2/PACSIN2 and EHD1, known members of tubular recycling endosomes and endosomal fission machinery. KD of LITAF/CDIP1 leads to an enhanced TRE network, again suggesting impaired endosomal fission. The authors provide evidence that LITAF is required for normal recycling of select receptors, such as integrins, but not others (ie., TfR). Localizations with TRE marked by Arf6, MICAL-L1, internalized CD59 and MHC1 point to roles in recycling of clathrin-independent cargo (consistent with the role of EHD1), and indeed, similar to EHD1 depletion, the loss of LITAF/CDIP1 caused a delay in cell migration into a wound by scratch assay. Overall, this is an interesting manuscript that provides significant new information on a key and under-studied biological process, the regulation of endosomal curvature and fission.

Reviewer 3 Comments for the Author:

This is a well-executed and significant study that will undoubtedly contribute to our current knowledge of endosomal regulation. In general, the experiments and data are robust, convincing and nicely demonstrated. Since the manuscript covers a wide range of functions and assays, there are several questions/points that would be helpful to address, but these critiques should not be misconstrued as negative, but rather suggestions either for this study or follow-up research.

Specific Comments/Critiques:

1) The authors might want to consider, either in the introduction or discussion, potentially addressing the role of Rab10 (and its interaction partners) in endosome biogenesis and/or fission. The recent studies from Etoh and Fukuda as well as Farmer et al. highlight the significance of Rab10 in TRE biology, and while it is understood that experimentally this is beyond the scope of the current study, addressing this and integrating it into the discussion might be helpful.

- We have included these important papers in the Introduction (line 61), and also alluded in the Discussion (lines 352-355) to the possible functional interaction between LITAF and microtubule motors in controlling the generation/scission cycle during TRE-dependent recycling.

2) While this reviewer recognizes that the relationship of LITAF to PI4P-containing membranes was not a major focus of the study (it appears only in the Supplemental data), this data could be strengthened in a variety of ways, as relying exclusively on anti-lipid antibodies can be problematic, due to the inherent issues with the quality of anti-lipid antibodies.

- The antibody is actually very well characterised, but we note the comment from the reviewer. We have now also included data using a PI4P sensor, which also colocalises extensively with the LITAF(SLD) tubules. See Figure S3D. See text lines 119-184)

© 2021. Published by The Company of Biologists under the terms of the Creative Commons Attribution License (https://creativecommons.org/licenses/by/4.0/).

3) Overlaying the LITAF fluorescence with a PM marker would be advisable for Fig. 1D

- With respect, we think the cell outline is quite clear and no PM marker is required.

4) The data from Supplemental Fig. 5F should be quantified.

- We have included quantification to support this figure panel. Supplemental Figure 5G, text line 244.

5) From Fig. 7, the authors infer impaired recycling of alpha5-integrins, and while that is likely the case, if possible it would be better to provide more direct evidence that measures recycling, rather than differences in subcellular distribution of the internalized protein.

- See also response to Reviewer 1. We agree that it is important to include a dynamic component to this experiment, rather than simply localising the integrin. We have therefore also quantitated the endosomal vs surface labelling of integrin at 30 minutes post uptake as well as at 120 minutes post uptake (see Figure 7B). These data show that surface labelling increases over time in the control cells (but not LITAF/CDIP1 KD cells) and thus represents integrin recycling.

6) It is recognized that this may be beyond the current (and quite wide) scope of the manuscript, but it would certainly be interesting to know whether LITAF interacts directly with EHD1, or whether it potentially interacts with Syndapin2/PACSIN2 or MICAL-L1 via proline-rich domains/SH3 domains, and that either/both of these proteins in turn interact with EHD1 through EH domain/NPF motifs.

- We agree that these are very important considerations, but feel that they are better addressed in a later study.

7) Another interesting question to address is whether LITAF recruits EHD1 to endosomes for fission (again, either directly or through common interactors).

- As above, our study certainly opens up several mechanistic questions about LITAF, but we feel these are better addressed in a focussed follow-up study.

<u>Second decision letter</u>

MS ID#: JOCES/2021/258549

MS TITLE: Endosomal recycling tubule scission and integrin recycling involve the membrane curvature supporting protein LITAF

AUTHORS: Lydia Wunderley, Ling Zhang, Rebecca Yarwood, Wenxia Qin, Martin Lowe, and Philip Woodman
ARTICLE TYPE: Research Article

I am happy to tell you that your manuscript has been accepted for publication in Journal of Cell Science, pending standard ethics checks.

© 2021. Published by The Company of Biologists under the terms of the Creative Commons Attribution License (https://creativecommons.org/licenses/by/4.0/).
